# Rice bacterial leaf blight drives rhizosphere microbial assembly and function adaptation

**DOI:** 10.1128/spectrum.01059-23

**Published:** 2023-10-17

**Authors:** Hubiao Jiang, Jinyan Luo, Quanhong Liu, Solabomi Olaitan Ogunyemi, Temoor Ahmed, Bing Li, Shanhong Yu, Xiao Wang, Chenqi Yan, Jianping Chen, Bin Li

**Affiliations:** 1 State Key Laboratory of Rice Biology and Breeding, Institute of Biotechnology, Zhejiang University, Hangzhou , China; 2 Department of Plant Quarantine, Shanghai Extension and Service Center of Agriculture Technology, Shanghai, China; 3 Taizhou Academy of Agricultural Sciences, Taizhou, China; 4 Ningbo Jiangbei District Agricultural Technology Extension Service Station, Ningbo , China; 5 Institute of Biotechnology, Ningbo Academy of Agricultural Sciences, Ningbo, China; 6 State Key Laboratory for Managing Biotic and Chemical Threats to the Quality and Safety of Agro-products, Institute of Plant Virology, Ningbo University, Ningbo, China; CCG-UNAM, Cuernavaca, Mexico

**Keywords:** rice bacterial leaf blight, rhizosphere microbiome, microbiome assembly, metagenomics

## Abstract

Rice bacterial leaf blight (BLB) is the most destructive phyllosphere bacterial disease caused by *Xanthomonas oryzae* pv. *oryzae*. The effect of soil-borne diseases on the rhizosphere microbiome has been extensively investigated. However, the regulatory role of the phyllosphere disease BLB on the rhizosphere microbiome remains unclear. Here, we observed that BLB significantly altered the composition of the bacterial-fungal community in the rhizosphere, decreasing bacterial diversity but not fungal diversity. The scale of inter-kingdom networks in the rhizosphere microbiome of BLB-infected plants was more complex and broader than in healthy plants, while the bacterial community was more vulnerable to BLB than the fungal community. Indeed, the relative abundance of *Streptomyces*, *Chitinophaga*, *Sphingomonas*, and *Bacillus* was higher in the BLB rhizosphere, which can be explained by their keystone hub taxa status in the rhizosphere co-occurrence network. Null model analysis showed that the deterministic assembly of bacterial communities increased while that of fungi decreased. Additionally, the assembly of bacterial and fungal communities was significantly related to variations in BLB and soil nutrients, especially pH and available phosphorus. Random forest model results showed that the bacterial community in the rhizosphere had a strong potential for predicting BLB. Metagenomic analysis revealed that BLB had a considerable impact on the functional adaptation of the rhizosphere microbiome. Interestingly, the abundance of some functional genes involved in carbon, phosphorus, and methane metabolism increased drastically.

**IMPORTANCE:**

Our results suggest that rhizosphere bacteria are more sensitive to bacterial leaf blight (BLB) than fungi. BLB infection decreased the diversity of the rhizosphere bacterial community but increased the complexity and size of the rhizosphere microbial community co-occurrence networks. In addition, the relative abundance of the genera *Streptomyces*, *Chitinophaga*, *Sphingomonas*, and *Bacillus* increased significantly. Finally, these findings contribute to the understanding of plant-microbiome interactions by providing critical insight into the ecological mechanisms by which rhizosphere microbes respond to phyllosphere diseases. In addition, it also lays the foundation and provides data to support the use of plant microbes to promote plant health in sustainable agriculture, providing critical insight into ecological mechanisms.

## INTRODUCTION

The interaction between the plant microbiome and the host plant plays a critical role in plant function and fitness in nature (1 – 5), which also serves as an essential factor in influencing plant health (6 – 8). Rhizosphere microorganisms contribute significantly to plant growth, nutrient acquisition, abiotic, and biotic stress (disease suppression) (9 – 15). The use of plant microbial communities to promote crop yields and manage plant diseases is a promising approach to sustainable agriculture (16 – 19). The plant rhizosphere ecosystem is affected by many variables, which can be categorized as biotic or abiotic, such as the host type (20, 21), soil type (22, 23), disease invasion, and insect damage (24).

In recent years, there has been increasing evidence that pathogen invasion affects microbial communities (25, 26). For example, the occurrence of different degrees of potato scab caused by *Streptomyces* spp. can significantly affect the microbial community composition and function of potato topsoil (geocaulosphere soil), demonstrating that specific inhibitory effects on pathogens were significantly enriched (25). Moreover, the rhizosphere and root interior of *F. graminearum*-infected wheat were significantly enriched for *Stenotrophomonas* (SR80), a beneficial bacterium that induces robust disease resistance by enhancing part of the plant’s defense responses (26). Recent research findings have documented that increasing the level of jasmonate in *Arabidops*is helps to recruit beneficial microbial communities to enhance plant disease resistance (27). The research carried out by Lee et al. (28) on tomato bacterial wilt discovered that the beneficial bacteria of rhizosphere microorganisms decrease bacterial wilt disease by stimulating the plant’s immune response (28). *Flavobacterium* (TRM1), which prevents tomato bacterial wilt, was isolated from the rhizosphere of soil resistant to tomato bacterial wilt (29). Hence, according to the above findings, it can be concluded that rhizosphere microbes contribute significantly to resistance to disease.

Rice (*Oryza sativa* L.) is the world’s most important food crop; however, *Xanthomonas oryzae* pv. oryzae, which causes bacterial leaf blight (BLB), one of the most important rice crop diseases, reduces its yield and consequently threatens food security (30). *Xanthomonas* is a Gram-negative bacterium and is one of the most important genera in plant bacteriology with 40 species of the bacteria able to infect approximately 400 plant species. It enters rice plants through wounds or water holes and settles rapidly on the xylem of leaves, hence, generating gray-brown lesions along the veins (31). The above-ground pathogen infection has been demonstrated to influence the plant rhizosphere microbial community (27, 32). However, the rhizosphere microbial community’s response mechanism to BLB remains unclear. As a consequence, we collected rice rhizosphere soils in five regions of Zhejiang Province with severe bacterial blight and performed amplicon sequencing (fungi and bacteria) and metagenomic sequencing. We systematically investigated the impact of phyllosphere diseases on the assembly and function of rhizosphere microbial communities through methods such as amplicon sequencing (fungi and bacteria) and metagenomic sequencing. This research report sheds new light on the microbial ecological mechanisms that rhizosphere microbial communities use to respond to BLB.

## MATERIALS AND METHODS

### Plant and sample collection

All samples used in this experiment were collected between 14 August and 16 September 2021. Healthy and diseased rhizosphere samples were collected from five regions namely: Jiangbei District, Ningbo City (121°32′54″E, 29°57′50″N), Yongjia County, Wenzhou City (120°44′14″E, 28°9′38″N), Ruian City (120°44′1″E, 27°50′22″N), Wucheng District, Jinhua City (119°25′10″E, 21°0′51″N), and Huangyan District, Taizhou City (121°17′19″E, 28°34′6″N). A total of 78 rice rhizosphere samples were collected. The diseased rhizosphere samples selected had rice plants showing severe symptoms of bacterial leaf blight, leaf chlorosis, and necrosis (Fig. S1), while the healthy samples showed no symptoms of rice bacterial blight (Fig. S1). In each region, at least six replicate samples were collected and each replicate sample was a mixture of three sub-samples. The rhizosphere samples of each rice plant from the field were collected, and the excess soil was shaken off as much as possible. The root system collected was kept in a 50 mL sterile centrifuge tube and stored in dry ice, which was transported to the laboratory and saved at −80 ℃ for further experiments. The disease index statistics (in terms of leaves) were conducted according to the grading standard of white leaf blight disease: grade 0: no disease, grade 1: spot area of less than 10% of leaf area, grade 3: spot area of 11 %–25 % of leaf area, grade 5: spot area of 26 %–45 % of leaf area, spot area of 46 %–65 % of leaf area, and spot area of leaf area of 65% or more. The calculation formula used is as follows: incidence rate = spot area/leaf area. Disease index = ∑ (number of diseased leaves at each level × relative level)/(total number of surveyed leaves × highest level value) × 100% (33).

### DNA extraction and amplicon sequencing

Microbial DNA was extracted from rice rhizosphere samples using the E.Z.N.A. Soil DNA Kit (Omega Bio-tek, Norcross, GA, USA) according to the manufacturer’s protocols. Bacterial and fungal sequencing libraries from 78 DNA samples were prepared. The V3–V4 region of the bacteria 16S ribosomal RNA gene was amplified by PCR (95°C for 2 min, followed by 25 cycles at 95°C for 30 s, 55°C for 30 s, and 72°C for 30 s, and a final extension at 72°C for 5 min) using primers 341F (5′-CCTAYGGGRBGCASCAG-3′) and 806R (5′-GGACTACNNGGGTATCTAAT-3′). The amplification of ITS1-ITS2 region (internal transcribed spacer) was performed using ITS1F (5′- CTTGGTCATTTAGAGGAAGTAA-3′) and ITS2R (5′- GCTGCGTTCTTCATCGATGC-3′) primers. PCR reaction was carried out using the following conditions: 30 s at 98°C; 35 cycles of denaturation at 98°C for 10 s, annealing at 54°C for 30 s, and extension at 72°C for 45 s; and a final step for 10 min at 72°C. Among them, the barcode was an eight-base sequence that was unique to each sample. PCR reactions were performed in triplicate of 20 µL mixture containing 4 µL of 5× FastPfu Buffer, 2 µL of 2.5 mM dNTPs, 0.8 µL of each primer (5 µM), 0.4 µL of FastPfu Polymerase, and 10 ng of template DNA. Amplicons were extracted from 2% agarose gels and purified using the AxyPrep DNA Gel Extraction Kit (Axygen Biosciences, Union City, CA, USA) according to the manufacturer’s instructions. Purified PCR products were quantified by Qubit 3.0 (Life Invitrogen) and every 24 amplicons whose barcodes were different were mixed equally. The pooled DNA product was used to construct the Illumina Pair-End library following Illumina’s genomic DNA library preparation procedure. Then, the amplicon library was paired-end sequenced (2 × 250) on an Illumina MiSeq platform Shanghai Biozeron Co., Ltd (Shanghai, China) according to the standard protocols.

### Physiochemical properties of the soil

Fresh soil was used for the determination of ammonium (NH_4_^+^-N) and nitrate (NO_3_^−^-N) content. NH_4_^+^-N and NO_3_^−^-N were extracted using 1 M KCl and their concentrations were measured in a continuous flow analytical system (AA3; Seal Analytical GmbH, Norderstedt, Germany). Soil pH was measured in soil:water solution (1:2.5) using a pH meter (Mettler-Toledo, Shanghai, China). Soil-available phosphorus (AP) was extracted with 0.5 M sodium bicarbonate (NaHCO_3_) at pH 8.5 and analyzed using the molybdenum antimony anticolorimetric method and an ultraviolet-visible spectrophotometer (UV-2600; Shimadzu, Kyoto, Japan). Available nitrogen (AN) was determined using the alkaline permanganate method (34). Briefly, HNO_3_ was used as the extract solution, and the soil sample was mixed in a ratio of 20:1, which was shaken for 0.5 hour and filtered immediately. The potassium in the solution was directly measured using a flame photometer, and soil total carbon and total nitrogen were determined using a Vario Max elemental analyzer (Vario Max, Elementar, Langenselbold, Hesse, Germany). Soil organic matter content was determined using the potassium dichromate volumetric method.

### Sequence processing

All analyses of sequencing data were performed in QIIME2 software (Quantitative Insights Into Microbial Ecology 2) (35). First, primers were cut using the cutadapt plugin (36). The DADA2 plugins were quality filtered, denoised, and chimeras were removed (37). Besides, taxonomy assignment was done to ASVs (amplicon sequence variants) using the classify learn naive Bayes classifier (38) based on the SILVA 132 (for bacteria) database and UNITE 8.0 (for fungi) database.

### Shotgun metagenomic sequencing and analysis

The metagenomic shotgun sequencing of rice rhizosphere samples was constructed and sequenced at Shanghai Biozeron Co., Ltd (Shanghai, China). All samples were sequenced in paired-end 150 bp (PE150) mode on an Illumina HiSeqX instrument, and raw sequence reads were quality trimmed using Trimmomatic to remove adapter contaminants and low-quality reads (39). Clean data were obtained by comparing the obtained clean sequences with the host plant genome by the BWA mem algorithm to remove host genome contamination and low-quality data. Clean reads were compared with all bacterial, archaeal, fungal, viral, protozoan, and algal genome sequences in the NBCI database using Kraken2 software (v2.1.1) (40). A clean set of sequence reads per sample was generated using MEGAHIT (41), followed by Prodigal (v2.6.3) (42) to predict the open reading frames (ORFs) of the assembled contigs, and all ORFs generated a set of non-redundant genes after clustering using CD-HIT (43). For gene annotation, BLASTX was used to search the the Kyoto Encyclopedia of Genes and Genomes (KEGG) database for unique gene sets to identify proteins to retrieve their functional annotations. According to the KO results of the samples, the specific functions and pathways of each sample were obtained using the annotated gene mapping pathways of the KEGG pathway database. Predicted genes were converted to amino acid sequences using DIAMOND (44) and compared with the Carbohydrate Active Enzymes (CAZy) database (45) and eggNOG database (46).

### Bioinformatics analysis

The beta nearest taxonomic index (βNTI) was calculated using the null model (999 randomizations) to assess the deterministic and stochastic processes of bacterial community assembly in the rice rhizosphere samples, where |βNTI| > 2 and |βNTI| < 2 represent the deterministic and stochastic processes, respectively. The bacterial community assembly process was further divided into five ecological processes based on βNTI and Bray-Curtis-based Raup-Crick Index (RC-Bray) values, namely βNTI > 2: homogenous selection, βNTI < −2: variable selection, |βNTI| < 2 and |RC-Bray| < 0.95: undominated processes, |βNTI| < 2 and RC-Bray <−0.95: homogenizing dispersal, and |βNTI| < 2 and RC-Bray > 0.95: dispersal limitation (47). To assess the main factors influencing the process of bacterial and fungal taxa assembly in healthy and diseased rhizospheres, Mantel tests based on Spearman’s correlation coefficients were performed to compare the Euclidean distance matrix and βNTI values for each variable.

The R package “Hmisc” and “graph” were used to calculate the rhizosphere microbial community co-occurrence network for healthy and diseased groups [relative abundance (RA) > 0.5%, significant correlation *P* < 0.05, Spearman coefficient *N* > 0.7 or <−0.7]. Also, network topology domain analysis was conducted based on average degree, average weighted degree, clustering coefficient, and closeness centrality. Visualization was performed using Gephi 0.9.2 (48). Correlations between rhizosphere microbial communities (bacterial and fungal-based ASVs) and soil physicochemical properties were assessed by Mantel tests. In order to obtain the best discriminative performance of taxa in the healthy and diseased rhizosphere, the Random Forest package was used. A total of 80% of the data were used as the training set, and the Random Forest (importance = TRUE, proximity = TRUE) was used as a function to generate a healthy and disease classification model. Cross-validation was performed by the rfcv () function to select appropriate features. The remaining 20% of the data were used as a test set for models with default parameters.

### Statistical analysis

All sequence data statistical analysis was mainly performed using the R package (v3.6.0). Non-parametric statistical tests were performed (Kruskal-Wallis test or Wilcoxon test) to assess alpha diversity and taxonomical differences at various stages. Based on Bray-curtis distances, the beta diversity of microbial community structure was compared by non-metric multidimensional scaling analysis (NMDS) and PERMANOVA (permutational multivariate analysis of variance) (49) to assess the significant differences of microbial community structure between groups. Analysis of enriched and depleted ASVs in infected and uninfected phyllosphere was done using the DESeq2 package. At the functional level, differentially abundant KO (KEGG orthologs), CAZy gene families, and Clusters of Orthologous Groups of proteins (COG) were identified using DESeq2. Wilcoxon and Kruskal-Wallis tests were used to examine statistical differences between treatments.

## RESULTS

### BLB affects rhizosphere alpha diversity and beta diversity

**Fig 1:**
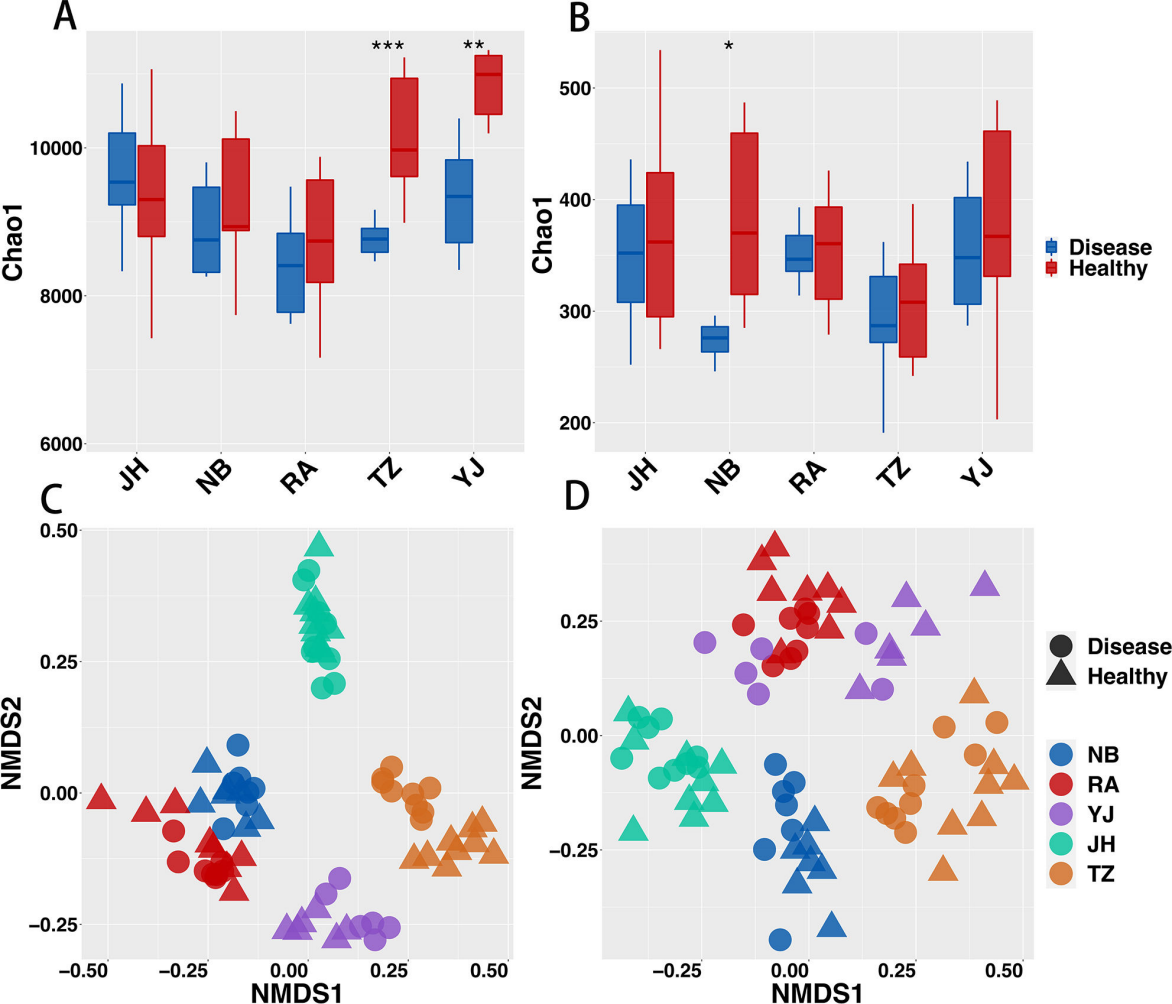
Alpha diversity and beta diversity of bacterial (**A, C**) and fungal (**B, D**) communities in the rhizosphere of healthy and diseased rice. Alpha diversity is characterized based on the Chao1 index. Significant differences were determined using the Kruskal-Wallis test, where asterisks indicate significant differences (*P < 0.05; **P < 0.01; and ***P < 0.001). NMDS is based on Bray-Curtis matrix analysis. Significant differences in communities were tested using PERMANOVA. NB: Ningbo samples, RA: Ruian samples, YJ: Yongjia samples, JH: Jinhua samples, and TZ: Taizhou samples.

From the 78 samples collected, 3,569,207 bacterial 16S rRNAs and 3,426,095 fungal ITS2 of high-quality sequences were obtained. These high-quality sequences were assigned to 10,176 fungal ASVs and 37,088 bacterial ASVs. Overall, the Chao1 index of the infected rice roots for bacteria was significantly reduced (Fig. S2A) with a more significant decrease in diversity observed at Taizhou (TZ) and Yongjia (YJ) (Fig. 1A). Fungi, on the other hand, did not exhibit any significant difference (Fig. 1B; Fig. S2B). The relative contributions of BLBs and sampling sites to the development of rhizosphere microbial communities were examined. The findings of the NMDS ranking analysis and the PERMANOVA analyses revealed that the sampling site had the greatest significant impact on the change of the rhizosphere microbiome (bacteria: *R2* = 0.54, *P* < 0.05, fungi: *R2* = 0.38, *P* < 0.05) (Fig. 1C and D; Fig. S4; Table S1). BLB contributed a higher percentage of change in rhizosphere fungal microbial communities than bacterial communities (bacteria: *R2* = 0.015, *P* < 0.05; fungi: *R2* = 0.023, *P* < 0.05) (Fig. 1C and D; Fig. S4; Table S1). Furthermore, the Bray-Curtis dissimilarity and difference index (Fig. S1C and D) was used to evaluate the similarity and the difference between the healthy and diseased rhizosphere microbial communities. It was observed that the microbial communities of the rhizosphere of diseased rice groups were more susceptible to change than the healthy groups. Conclusively, BLB had a substantial impact on the structure and composition of rice rhizosphere microbial communities.

**Fig 2:**
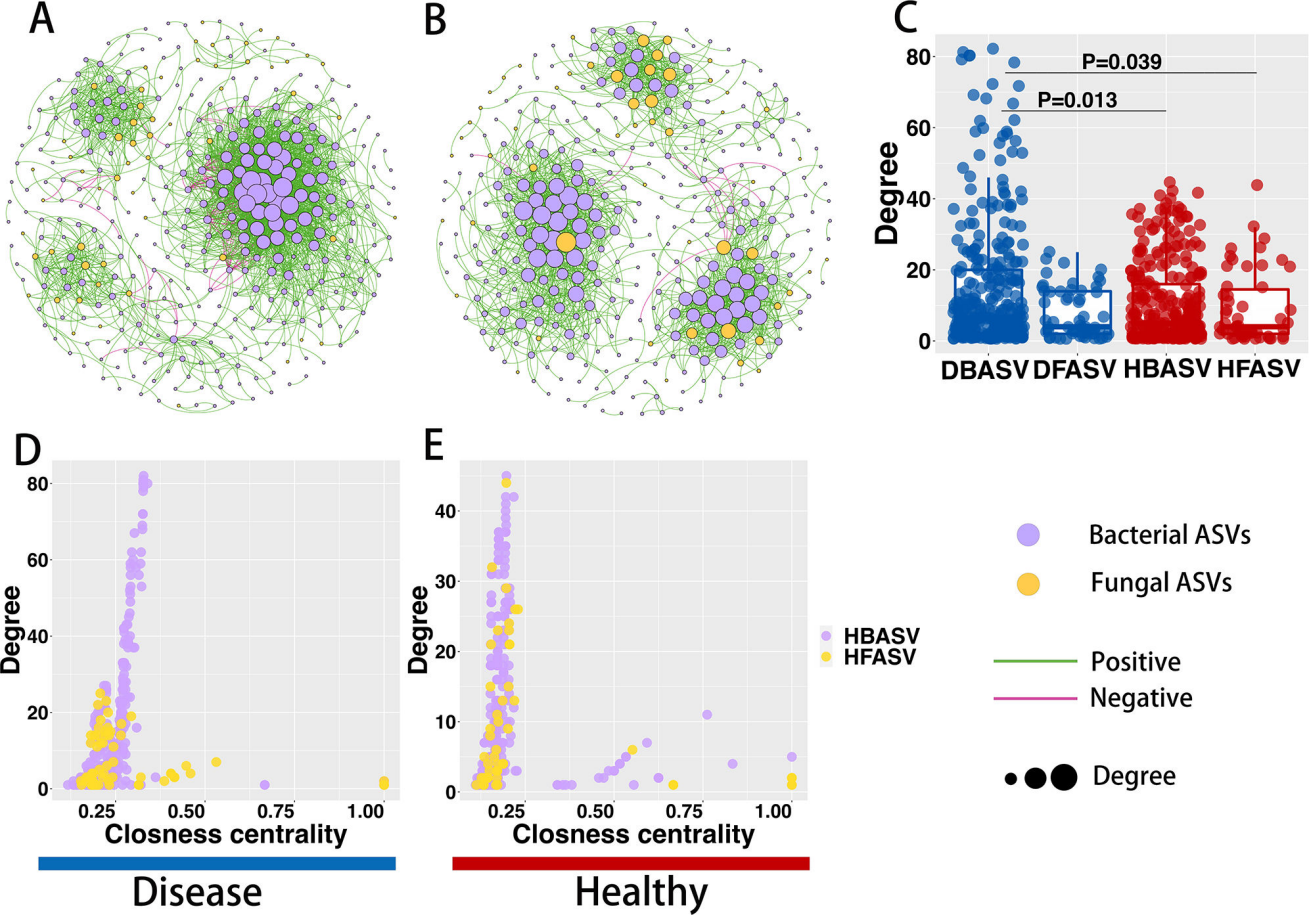
Co-occurrence networks between fungal and bacterial kingdoms in diseased (**A**) and healthy (**B**) rhizospheres. (**C**) Degree values of bacterial and fungal taxa in healthy and diseased networks. The significant difference was determined by non-parametric Kruskal-Wallis test. Diseased (**D**) and healthy (**E**) comparison of networks’ node-level topological features (degree and closeness centrality). Node size indicates the network degree. Each node represents an ASV, purple represents bacterial ASV, and yellow represents fungal ASV. Correlations are indicated between nodes (correlation coefficient > 0.7 and the green line indicate a positive correlation; correlation coefficient < −0.7 and the red line indicate a negative correlation).

Mantel analysis was used to investigate the relationship between soil physical and chemical properties (Table S5) and to consider whether the changes in the soil physicochemical properties influenced the structural differentiation of microbial communities. The analysis revealed that the bacterial community in the diseased group was strongly correlated with AK, while the fungal community was strongly correlated with NO_3_^−^-N and AP. Hence, a strong correlation between the bacterial and fungal communities and the AP content in the healthy group was observed (Fig. S3).

### BLB affects rhizosphere microbial community co-occurrence networks

A co-occurrence network analysis was performed to assess the effect of BLB on inter-microbial-fungal-bacterial kingdom interactions in the rice rhizosphere niche. According to our findings, the fungal-bacterial inter-kingdom network pattern changed significantly between two separate ecological niches; healthy and diseased. This revealed that the underlying interactions of the fungi and bacteria differed in the healthy and the diseased rhizosphere microbial networks (Fig. 2A and B). The average degree (13.371), number of edges (2,507), and number of nodes (375) observed in the diseased network were higher than those in the healthy network (average degree: 10.072, number of edges: 1,813, and number of nodes: 360) (Fig. 2C, D, and E). The diseased fungal-bacterial kingdoms exhibited lower network modularity and average path length than the healthy network. However, the inter-kingdom networks of bacteria and fungi showed the reverse pattern (Fig. S5 and S6; Table S2). Additionally, the number of nodes and edges of the bacterial taxa in the diseased network was higher than those in the healthy network, while the reverse pattern was observed in the fungal taxa (Fig. S5 and S6). Bacterial-fungal inter-kingdom networks were 99.5% positively correlated to the healthy group and 96.6% positively correlated to the diseased group. The bacterial intra-kingdom networks were mostly positively correlated (healthy group: 99.6%, diseased group: 96.4%), while the fungal intra-kingdom networks were all positively correlated (Fig. S6; Table S2).

Network keystone taxa are defined as nodes with a high degree (degree > 20) and closeness centrality (closeness centrality > 0.2) in the network. The diseased network comprised 87 network keystone hubs with fungi having 4 network keystone hubs and bacteria having 83 network keystone hubs. These network keystone hubs were primarily composed of *Acidobacteria*, *Chloroflexi*, *Firmicutes*, *Gemmatimonadetes*, *Proteobacteria*, *Mucoromycota*, *Microsporidia*, *Chytridiomycota*, and others (Table S3). Specifically, the diseased-bacteria-fungus inter-kingdom network keystone hubs mainly comprised bacteria like *Streptomyces*, *Sphingomonas*, *Bacillus*, *Gemmatimonas*, and *Cronobacter*, as well as fungi such as *Rhizophydium*, *Mortierella*, and *Diversispora* (Table S3). However, the number of keystone hub species in the diseased rhizosphere co-occurrence network of bacteria (50) was higher than that of the healthy rhizosphere (49), while the number of the keystone hubs in the diseased rhizosphere co-occurrence network of fungi (28) was less than that of the healthy rhizosphere (34) (Table S3).

### BLB affects taxonomic composition in rhizosphere communities

**Fig 3:**
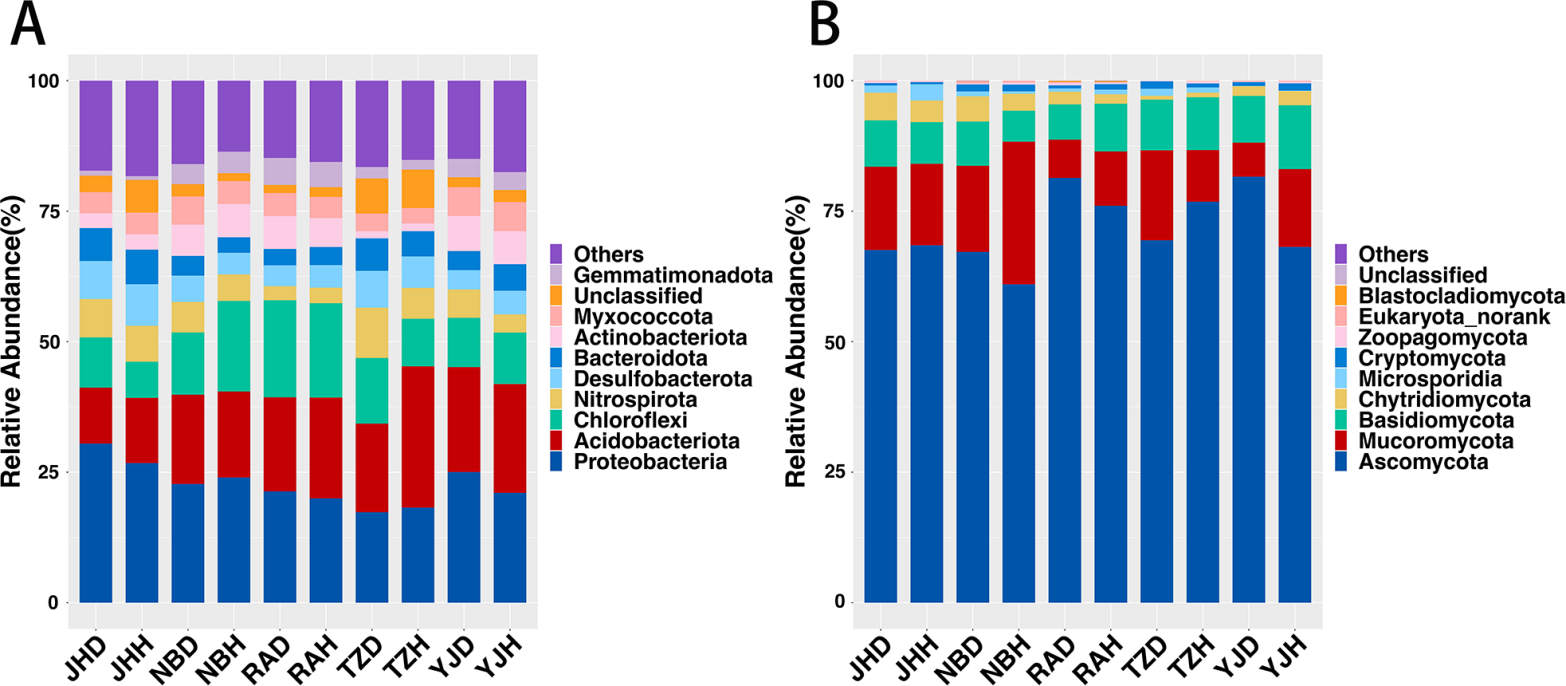
Taxonomic composition of the rice rhizosphere microbial community. RA of different bacteria (**A**) and fungi (**B**) in healthy and diseased. The bacteria or fungi ranked outside the TOP10 were grouped into “Others.” NBD: Ningbo diseased samples, NBH: Ningbo healthy samples. RAD: Ruian diseased samples, RAH: Ruian healthy samples. YJD: Yongjia diseased samples, YJH: Yongjia healthy samples. JHD: Jinhua diseased samples, JHH: Jinhua healthy samples. TZD: Taizhou diseased samples, and TZH: Taizhou healthy samples.

BLB affected the bacterial and fungal composition of the rhizosphere community. The bacteria in all the rhizosphere samples were mostly *Proteobacteria*, *Acidobacteriota*, *Chloroflexi*, *Nitrospirota*, *Desulfobacterota*, *Bacteroidota*, *Actinobacteriota*, *Myxococcota*, Unclassified, and *Gemmatimonadota,* which accounted for 81.7%–86.4% phyla (Fig. 3A). *Ascomycota*, *Mucoromycota*, *Basidiomycota*, *Chytridiomycota*, *Microsporidia*, *Cryptomycota*, *Zoopagomycota*, and *Eukaryota* had no rank and were the most abundant fungi, which accounted for more than 99.9% of all microorganisms (Fig. 3B). The differences in the abundance of bacteria and fungi in the rhizosphere microbes of healthy and diseased rice groups were further compared by DEseq2. The results showed that there were 91 bacterial ASVs (47 ASVs enriched and 44 ASVs depleted) in the rhizosphere, which were significant, while fungi had 30 ASVs that were significantly different (13 ASVs enriched and 17 ASVs depleted) (Fig. S7). Bacteria such as *Rhodanobacter*, *Streptomyces*, *Escherichia*, *Nitrososphaera*, and others, as well as fungi, which mostly included *Nigrospora*, *Slooffia*, *Epicoccum*, and others, were widespread (Fig. S7B). Moreover, further analysis by region revealed that the abundance of *Streptomyces* was significantly increased in the diseased rhizospheres of the four regions (Fig. S7A).

### Rhizosphere bacterial and fungal community assembly

**Fig 4:**
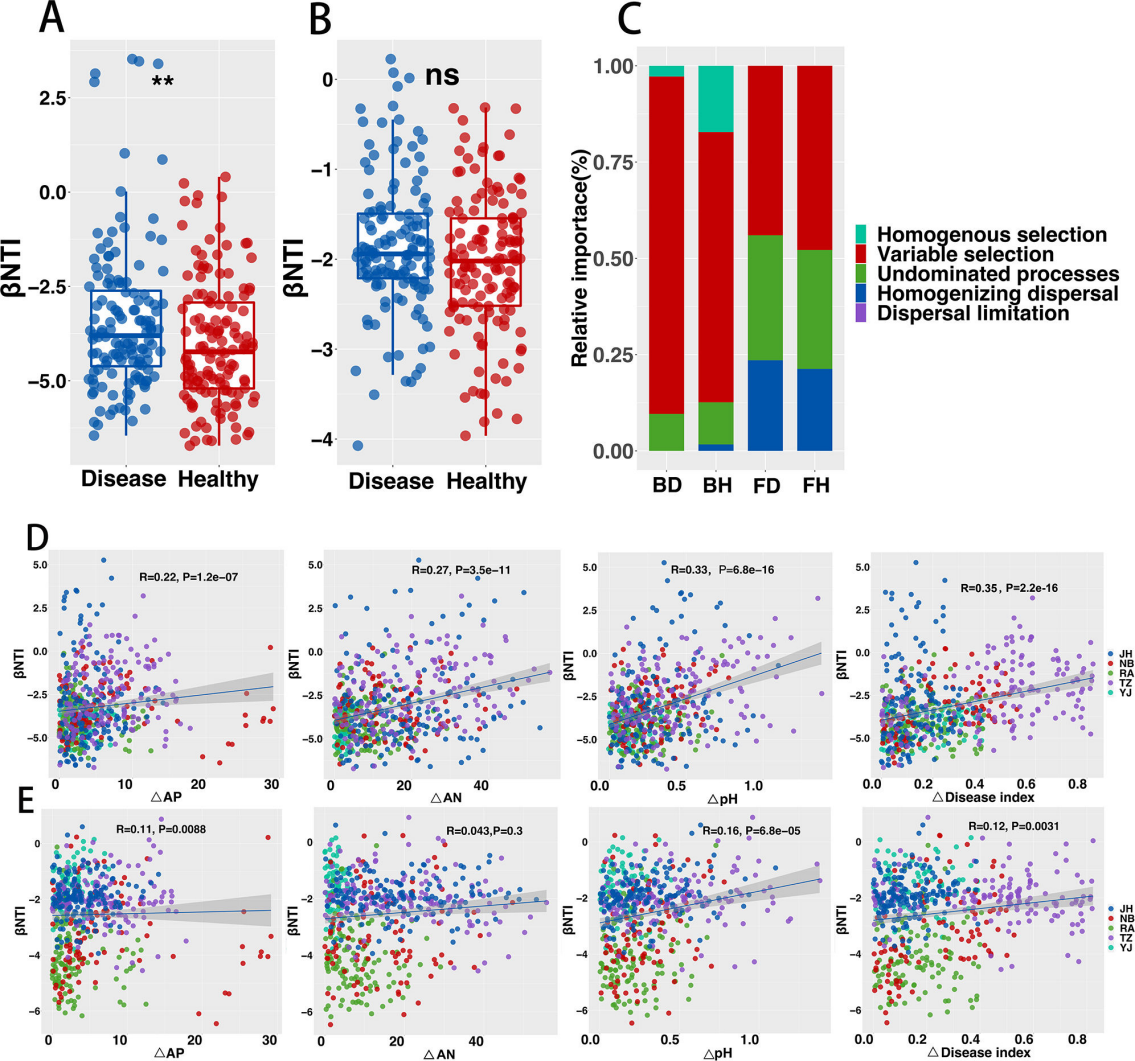
Assembly processes of healthy and diseased rhizosphere microbial communities (bacteria and fungi). The weighted βNTI of bacterial (**A**) and fungal (**B**) communities. (C) Relative importance of bacterial and fungal community assembly processes. Spearman correlations between βNTI and changes in environmental variables for bacterial (**D**) and fungal (**E**) communities. The gray dotted lines indicate the upper and lower thresholds for βNTI = 2 and −2, respectively. Asterisks indicate significant differences (**P < 0.01) and ns indicates no significant differences.

To quantify the deterministic and stochastic processes of microbial community assembly, a combination of βNTI and RC-Bray was used. Variable selection of 70.16% in the deterministic process was primarily responsible for a healthy rhizobacteria microbiome (Fig. 4). In comparison, the fungal community was primarily determined by deterministic variable selection of 47.83%, stochastic undominated processes of 30.89%, and homogeneous selection of 21.28%. (Fig. 4C). The bacterial community in the diseased rhizosphere was primarily determined by deterministic variable selection (87.62%), while the fungal community was determined by variable selection of deterministic processes (44.02%), stochastic undominated processes (13.89%), and homogeneous selection (52.78%). Overall, the bacterial community in the healthy rhizosphere was determined primarily by deterministic process (Fig. 4A), while the fungal community was determined by both deterministic and stochastic processes (Fig. 4B). Nevertheless, the diseased groups showed a similar picture. The increased relative importance of deterministic rhizosphere bacterial communities in the diseased group was significant, whereas the relative importance of the fungal deterministic process decreased and the relative importance of stochastic process increased. Furthermore, the disease rhizosphere bacteria in the five regions were mainly determined by the variable selection (66.67%–100%) of the deterministic process. The fungi in the diseased rhizosphere are primarily determined by the variable selection (33.33%–50.00 %) of the deterministic process, the stochastic undominated processes (13.89%–47.62 %), and homogeneous selection (9.52%–52.78 %) (Fig. S8). Furthermore, correlation analysis showed that the bacterial community assembly in the rhizosphere was significantly positively correlated with changes in AP, AN, and pH (Fig. 4E), while the fungal community was significantly negatively correlated with AP (Fig. 4F), and AN was irrelevant. Thus, it should be noted that the disease index was positively correlated to community assembly (Fig. 4E and F). Therefore, this indicates that disease and soil nutrition both had a significant impact on the assembly of the rhizosphere bacterial and fungal communities.

### Rhizosphere bacterial communities as biomarkers of health and disease

**Fig 5:**
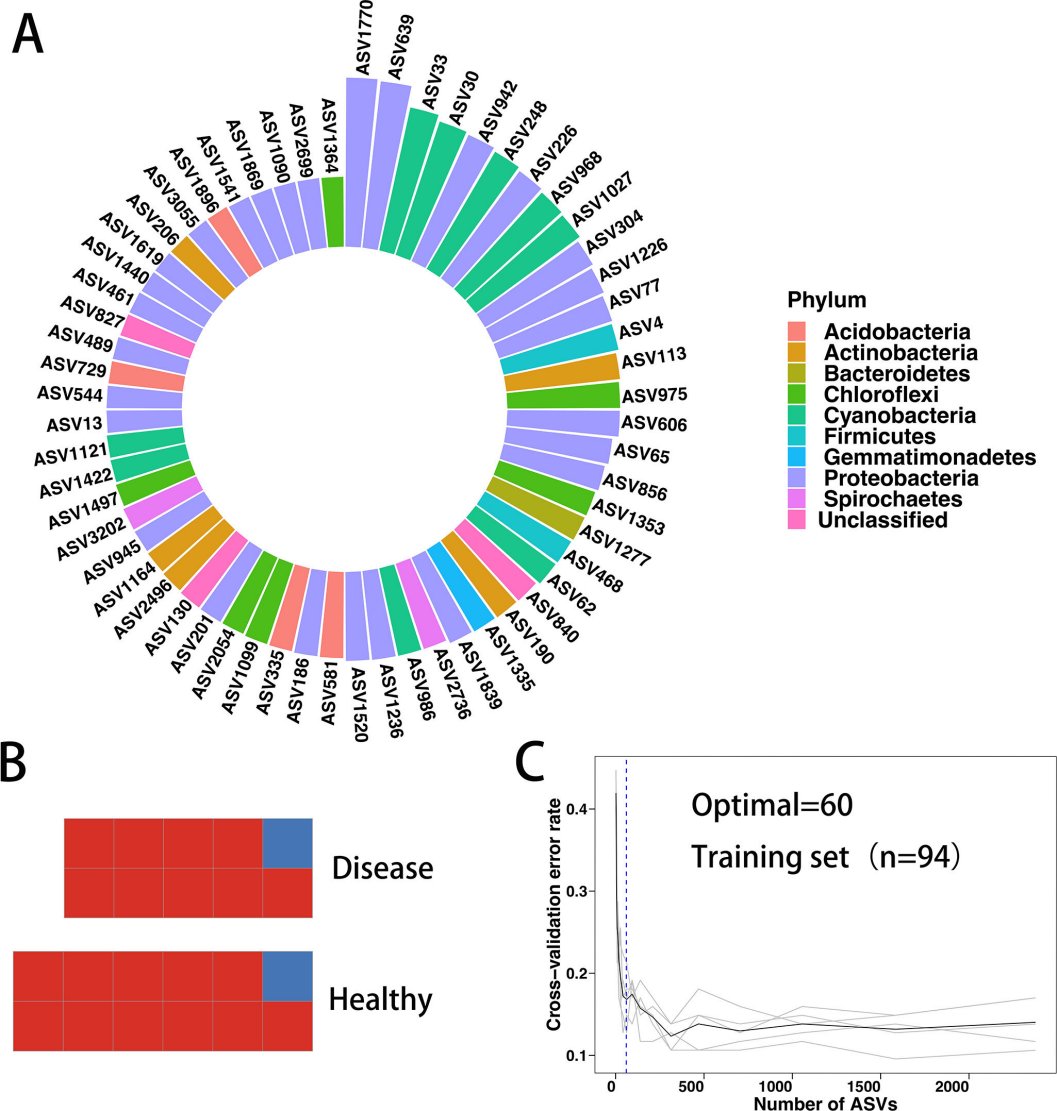
Random-forest model detects bacterial taxa that accurately predict health and disease. The top 60 biomarkers of importance in the random forest classification model. Using random forest classification, all healthy and diseased rhizosphere microbiomes (94 samples in total) were classified at the ASV level and colored at the phylum level. Biomarker taxa were listed in descending order of importance to model accuracy. (B) Results of the predictions based on the random forest model for healthy and diseased rhizosphere samples were not included in the training set. Each square represents a sample in the test sample. (C) 10-fold cross-validation error as a function of the number of input taxa was used to distinguish between the healthy and diseased root microbiota ordered by importance.

We investigated whether the rhizosphere microbial community membership could be used as a biomarker to distinguish between healthy and diseased rice plants. To do this, we constructed a random forest machine learning model that related healthy and diseased groups with rhizosphere microbial community data at the phylum, class, order, family, genus, and ASV levels. The accuracy of this bacteria model for rhizosphere microbiota classification was 87.2%, which was the highest among all taxonomic levels. To assess the importance of the indicator microbes, 10-fold cross-validation with five replicates was employed. When 60 significant taxa were used, the cross-validation error curves stabilized (Fig. 5A and C), and when the top significant clusters were chosen, the number of clusters against the cross-validation error curve was very stable. As a result, we categorized these 60 as biomarker clusters. The RA of *Enterobacteriaceae*, *Nostocaceae*, *Micrococcaceae*, and other microorganisms in the diseased rhizosphere was relatively high (Fig. S9). *Acinetobacter* (ASV1770) was the most important biomarker among them (Fig. 5A). However, the average accuracy rate of the test results of the 24 samples that did not participate in the model training in this study was 91.7%, where only two samples were wrong (Fig. 5B). In conclusion, our results indicate that there are consistent differences in the rhizosphere microbiome of healthy and diseased rice plants. These differences can be used to train a random forest machine learning model that can predict whether a rice plant is healthy or diseased with high accuracy. This finding has important implications for the development of new strategies for preventing and controlling BLB.

### BLB affects rice rhizosphere microbiome function

**Fig 6:**
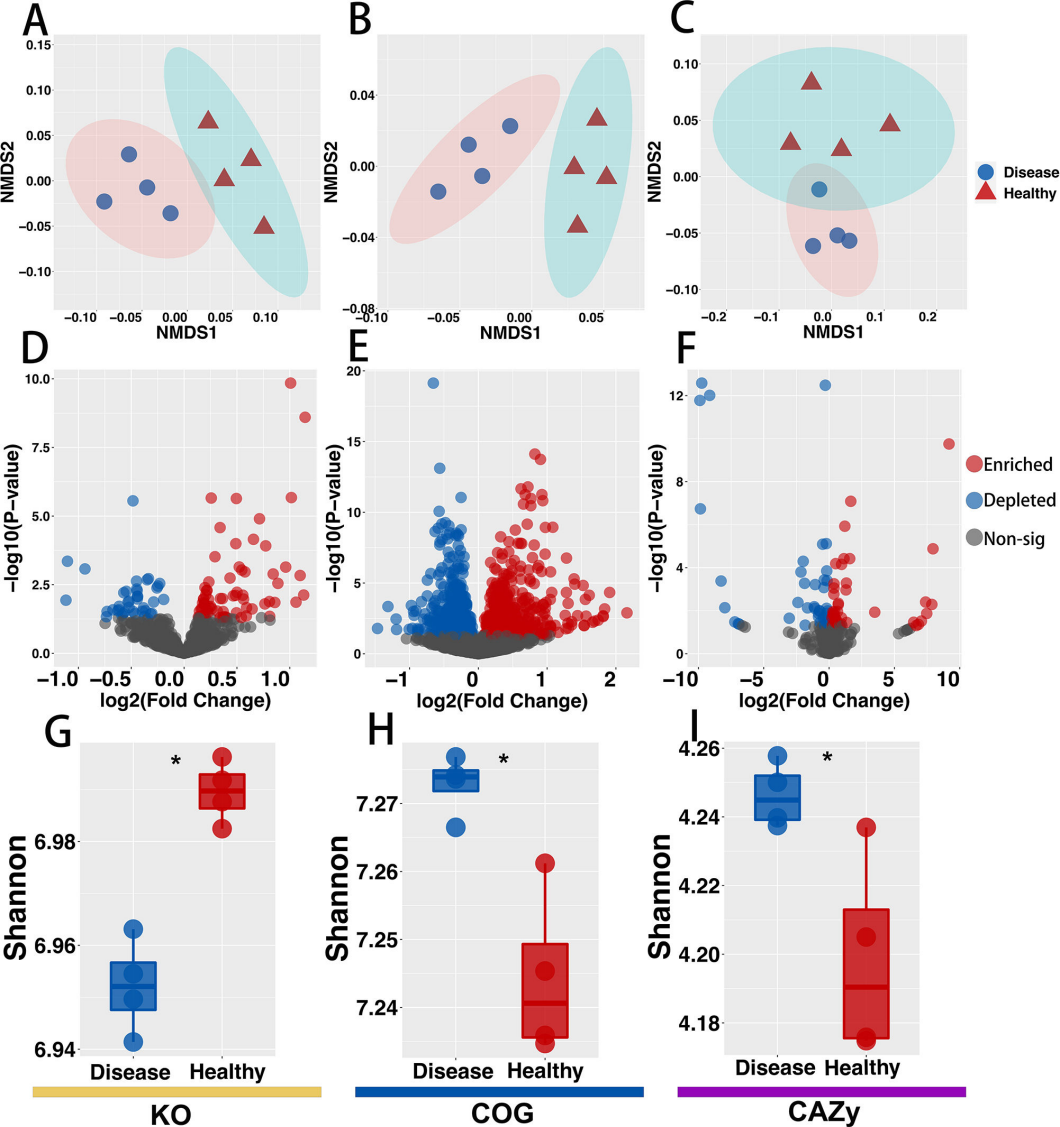
Functional diversity and differential changes in healthy and diseased rhizosphere microbiomes based on KO, CAZy, and COG functions. KO (**A**), COG (**B**), and CAZy (**C**) NMDS ranking of functional genes based on Bray-Curtis distance matrix. Volcano plots show enrichment and depletion patterns of healthy and diseased rhizosphere microbes based on KO (**D**), COG (**E**), and CAZy (**F**). Boxplots show the functional alpha diversity of the microbiome based on KO (**G**), COG (**H**), and CAZy (**I**) functions. Alpha diversity was based on the Shannon index. Asterisks indicate significant differences (*P < 0.05).

Metagenomic analysis was used to evaluate the functional alteration of the rice rhizosphere microbiome caused by BLB. Samples from the TZ region were selected for metagenomic sequencing. Metagenomes were assigned to 30,229 bacteria and 630 fungi. The functional composition of the rhizosphere microbiome differed substantially between healthy and diseased rhizosphere microbiomes, as evidenced by KEGG Orthology, COG, CAZy, and NMDS ranking of Comprehensive Antibiotic Resistance Database (CARD) (Fig. 6A, B, and C; Table S1). Notably, the BLB rhizosphere microbiome was established on KO. Although the functional diversity of BLB decreased (Fig. 6G), functional diversity based on COG and CAZy was higher in the healthy group (Fig. 6H and I).

According to the KEGG database comparison results, 68 KOs were significantly enriched and 39 KOs were depleted in the BLB rhizosphere. The 68 enriched KOs were mostly involved in carbohydrate metabolism (18), cofactor and vitamin metabolism (10), membrane transport (8), energy metabolism (8), amino acid metabolism (6), nucleotide metabolism (4), exogenous biodegradation and metabolism (4), and plant interaction (Fig. 6D). By comparing the rhizosphere microbiome of the diseased group to the CAZy database, it was observed that the diseased group of rhizosphere microbiome had higher CAZy diversity than the healthy group (Fig. 6I). Differential enrichment findings showed that 41 CAZy families were significantly enriched in the diseased rhizosphere, while 42 CAZy families were significantly depleted (Fig. 6F). The 41 CAZy discovered in the rhizosphere belonged to glycoside hydrolases (21), carbohydrate-binding modules (10), glycosyltransferases (6), and polysaccharide lyases (4). It is worth noting that amylase (beta-amylase) was the diseased group with the highest significant increase (Fig. 6E). This indicates that the diseased rhizosphere microbiome is more capable of behaving as a glycoside hydrolase than healthy ones. Furthermore, when compared to the eggNOG database, 445 COG functional proteins were shown to be significantly increased in the diseased rhizosphere, while 404 COG functional characteristics were decreased. Notably, the most abundant enriched functional protein was Cellular Processes and Signaling (COG_T) (Fig. 6F).

## DISCUSSION

In the present study, the rice rhizosphere microbiome response to BLB was explored using amplicon and metagenomic approaches. The rhizosphere microbiome is well-known to play an important role in plant stress resistance and disease suppression, improving plant tolerance and resistance to environmental stress (51 – 53). Previous studies have shown that the alpha diversity of rhizosphere microbial communities plays a major role in plant stability and adaptability to stress and invasion. This is because a highly diverse microbial community is more likely to have the necessary traits to resist stress and invasion. Additionally, a diverse community is more likely to be able to adapt to changes in the environment (54 – 56). However, our findings revealed that the alpha diversity of the diseased rhizosphere bacterial community was lower than that of the healthy rhizosphere (Fig. 1A; Fig. S2A), whereas there was no significant difference in the fungal communities (Fig. 1B; Fig. S2B), which is similar to previous studies (57, 58). Furthermore, our results revealed that the beta diversity of bacterial and fungal communities in the diseased rhizosphere was much lower than that observed in the healthy rhizosphere. In addition, the sampling site had a greater impact on community composition than BLB (Fig. 1C and D; Table S1). Re-analysis of samples from different regions found that BLB can significantly affect the rhizosphere microbial community composition (Fig. S2A, B and S4; Table S1), which indicates that BLB can indeed significantly affect the rhizosphere microbial community structure. Taken together, these findings suggest that BLB significantly decreased the diversity of bacterial communities in the rhizosphere, with a greater impact on the bacterial community than the fungal community.

Co-occurrence network analysis provided novel insights into the complex microbial communities and the interactions within microbial communities. In addition to the differences in bacterial communities’ diversity, diseased rhizospheres formed microbial co-occurrence networks with a higher number of edges, nodes, and average degree than healthy plants. However, fungal communities had the opposite pattern (Fig. 2; Fig. S5; Table S2). When microbes are stressed, highly connected co-occurrence networks could form (59) and such complex networks are more favorable to plant growth, hence, enhancing their adaptability to environmental shocks than simple networks. Wei et al. (57) reported that the complexity of the community co-occurrence network in the cotton rhizosphere increased significantly after *Verticillium dahlias* infection (57). In agreement with this study, the bacterial co-occurrence network had a higher proportion of negative edges than the fungal network (Fig. S5; Table S2). Contrary to popular opinion that mutual negative in microbial networks suggests highly intense ecological competition, and the uncertainty caused by such competition will promote network stability and the host thereby benefits from the microbial competition (60). Furthermore, because modularity is strongly associated with network stability, the low modularity of bacterial communities in the diseased rhizosphere exacerbates network instability (61). This was also revealed in this study in that the bacterial community in the rhizosphere was more sensitive to BLB than the fungal community. Our findings suggest that BLB increased the complexity of bacterial communities while having less influence on fungal networks. Hence, it can be concluded that bacterial communities were more susceptible to BLB.

A substantial difference was observed in the microbial community composition between the BLB rhizosphere and the healthy rhizosphere in terms of species composition (Fig. 3). Potentially beneficial bacteria such as *Streptomyces*, *Chitinophaga*, *Sphingomonas*, and *Bacillus* were more abundant in the BLB rhizosphere (Fig. S7). It has been reported that when plants get infected with pathogens, plants release root secretions (such as amino acids, long-chain organic acids, and so on) in order to attract beneficial microbes that will help the plants combat external environmental stress and boost disease resistance (27, 62 – 64). Many previous studies have reported that *Streptomyces*, *Chitinophaga*, *Sphingomonas*, and *Bacillus* played an important role in regulating plant physiological states, especially in disease mitigation (15, 65 – 68). *Streptomyces*, for example, has the capacity to synthesize bioactive chemicals, antibiotics, and extracellular enzymes. It could even contribute to disease resistance by producing antibiotics and siderophores, volatile secretions, and partake in nutrient competition (28, 65, 69). Also, it has been reported that *Streptomyces* can directly promote plant growth by producing phytohormones and enhancing nitrogen fixation (50, 70). *Sphingomonas*, on the other hand, was significantly enriched in the citrus phyllosphere, which enhanced the inhibitory effect on the pathogen, *Diaporthe citri*, through iron competition (66). Similarly, the genus *Bacillus* is a well-known biocontrol bacteria that protect plants from pathogenic bacteria through several mechanisms (such as antibiotic chemical synthesis, biofilm formation, and nutritional niche competition), which is extensively employed in plant disease control (71). Our findings, therefore, imply that when rice is challenged with BLB, the plants select some beneficial bacteria in the rhizosphere. *Streptomyces*, *Sphingomonas*, *Bacillus*, and other genera were considered as the keystone hub taxa of the co-occurrence network through network core species identification (Fig. 2; Table S3). Keystone hub taxa occupy key network topological positions and can be used to construct beneficial plant microbiomes (72). At the same time, these keystone hub taxa play a vital role in network stability (73). Furthermore, more beneficial bacteria that fall in the crucial groups play a core role in promoting plant growth after disease encounters (74). Our studies highlight the importance of these microbial taxa in the rhizosphere network. At the same time, it provides critical evidence for the plant’s “cry for help” strategy, which can better harness its microbiome to improve disease control. In the future, it will be necessary to isolate and culture these beneficial bacteria to determine their disease resistance and its molecular mechanism.

According to metagenomic data, BLB significantly reduced the functional diversity of KOs in the rhizosphere microbiome. A decrease in microbial diversity explains the decrease in functional diversity observed. However, the functional diversity of COG and CAZy increased significantly (Fig. 6G, H, and I). Furthermore, it was observed that the functional genes associated with plant-pathogen interaction (K02358, elongation factor Tu) were significantly enriched in the diseased rhizosphere. Different biases have been reported in shotgun metagenomic sequencing (75, 76). Hence, for further research on the significance of tuf enrichment, the changed gene could be sourced from the plant genome, to avoid bias in rice genome filtering. The functional diversity and enrichment in diseased and healthy rhizosphere microbiomes were shown by the metagenomic data. In terms of the functional characteristics, there were significant differences between the diseased and the healthy rhizospheres (Fig. 6). BLB lowered KO functional diversity significantly. A decrease in the rhizosphere microbial diversity might explain the decrease in functional diversity observed. Several studies have also reported that species diversity was critical for ecological function (77 – 80). A more diverse microbiome ensures that plants engage more extensively in ecological processes. Microbial communities having a high diversity are more stable and have more functional redundancy (81). Various functional categories related to microbial metabolism were significantly enriched in the diseased rhizosphere when compared to the healthy groups (Table S4), such as carbohydrate metabolism (18), cofactor and vitamin metabolism (10), membrane transport (8), energy metabolism (8), and so on.

This result, however, suggests that BLB strengthened the metabolic pathway-based interactions. Among them, the mannose PTS system (K02793) (Table S4), which is a bacterial PTS found only in bacteria and is responsible for transporting and catalyzing the phosphorylation of extracellular carbohydrates and provides various carbon sources for bacterial metabolism (82), was significantly enriched in the diseased rhizosphere of this study. Furthermore, some fructose bisphosphate aldolases (FBA, K01624) were found to be significantly enriched in the rhizosphere (Table S4). The FBA acts as an ATP source to power ion transport, helps the cyanobacterial cells to enhance their tolerance to salt stress, and regulates several metabolic pathways, as these metabolites originate from glycolysis (83). For example, CII-FBA (sll0018) was shown to increase growth rate and biomass accumulation in *Synechocystis* PCC 6803 cells (83). Notably, 4-hydroxy2-oxopentanoic acid aldolase (mhpE, K01666) was significantly enriched (Table S4). This MhpE protein pair has been reported to be essential for siderophore formation (84). In addition, a significant enrichment of the phosphonate transport system substrate-binding protein gene (phnD, K02044) was observed (Table S4), which enhances phosphorus metabolism, in addition to promoting carbon metabolism. It has been shown that targeting the phosphonate ABC transporter substrate-binding protein PhnD enhanced phosphate absorption and biofilm synthesis (85). Furthermore, livH (K01997), an ABC transporter substrate-binding protein, was significantly enriched (Table S4), and livH from *Rhizobium leguminosarum* bv *viciae* can promote N element fixation to regulate plant growth (86).

Furthermore, it was discovered that the relative abundance of the alkaline phosphatase gene phoD (K01113), which is responsible for organophosphate cycling, was reduced in the diseased rhizosphere (Table S4), thus indicating that BLB affected the host phosphorus absorption (87). Despite that, the health and diseased functional enrichment of the rhizosphere microbiome provided evidence for a plant “cry for help” strategy. However, this evidence should be validated through *in vitro* culture experiments with the aim to isolate enriched strains and study their disease resistance and underlying mechanisms. Furthermore, the proposed molecular mechanism for the increased abundance of plant-specific genes in response to biotic stress needs additional investigation. These findings imply that BLB affected the functional taxonomic characteristics of the rice rhizosphere microbiome. Diseases have previously been shown to have a considerable influence on plant microbiome assembly (25, 58). Our results indicate that disease affected the assembly and function of the plant rhizosphere microbiome.

Rice agriculture generates about 7%–17% of worldwide CH4 emissions, which are produced by anaerobic archaea in the rice rhizosphere (88). According to our findings, BLB rhizosphere tetrahydromethanopterin S-methyltransferase subunit H (mtrH, K00584) was considerably enriched in the rhizosphere (Table S4), and the subunit of tetrahydromethanopterin S-methyltransferase was involved in an essential enzyme for methane formation from CO (89). Furthermore, three formylmethanefuran dehydrogenase subunits (fmdA, K00200, fmdB, K00201, fmdC, K00200) were significantly enriched (Table S4). Formylmethanefuran dehydrogenase engages in the first step of the methane hydrogenotrophic pathway, the reduction of CO_2_ to formate in the first step of acetic acid generation. Methanogenic formate dehydrogenases oxidize formate to CO_2_ in supplying CO_2_ for hydrogenotrophic methanogenesis (90, 91). CdhD (K00194) and CdhE (K00197) were also shown to be significantly enriched in the BLB rhizosphere (Table S4). CdhD-CdhE are essential steps in the carbon fixation from CO to generate CH_4_. In other words, a corrinoid iron-sulfur complex is generated in order to provide a methyl group for the second step reaction (92). Taken together, our findings suggest that BLB may enhance the metabolic activity of archaeal methanogens in the rhizosphere microbiome. Hence, this work significantly extends our understanding of disease-induced rhizosphere microbiome functional assembly.

We combined βNTI with RC-Bray to determine how healthy and diseased bacterial and fungal assembly affects fundamental processes. Four fundamental processes (diversification, dispersal, selection, and drift) can be promoted in the assembly of microbial communities (93), which are employed to describe microbial assembly processes in various environmental scenarios (94 – 96). Our findings indicated that deterministic variable selection drives diseased bacterial community assembly, whereas stochastic non-dominant processes with homogenous dispersal drives fungal community assembly (Fig. 4). This shows a substantial microbial selection pressure among diseased rhizosphere bacterial communities, whereas fungi did not. The increased relative importance of deterministic processes to the assembly of bacterial communities could be attributed to increased environmental selection pressure and biotic interactions. For abiotic variables, it was found that the rhizosphere’s physicochemical properties, especially AK, AN, and pH, were significantly positively correlated with the assembled communities of bacterial and fungal communities (Fig. 4; Fig. S8), which indicate that these soil nutrients were likely to pass through in the community. Increased selective pressure on the rhizosphere bacterial survival and fitness is regulated by abundant nutrients. Furthermore, when plants are afflicted with pathogens, they will produce metabolites that regulate the activity of the rhizosphere microbial community, and then select the enrichment of beneficial microorganisms in the rhizosphere to manage the community’s assembly. Previous studies have shown that plants drive microbial community assembly through the enrichment of rhizosphere metabolites (tocopheryl acetate, citrulline, galactitol, stearylglycerol, and behenic acid) (97). There is also dynamic root exudate distribution that participated in the assembly of wild oat (*Avena barbata*) microbial communities (52). Finally, the existence of BLB may have played a role in the formation of the rhizosphere bacterial community.

Significant differences were observed in the rhizosphere communities between the healthy and diseased (Fig. 5); also, the influence of sample location on the rhizosphere community was considerably larger than the effect of BLB on the rhizosphere. Nonetheless, we were able to identify common features of bacterial populations in BLB rhizosphere soils using the RF model. The RF model applied in this study has had a widespread application in microbial ecology research (98 – 100). For example, microbial signatures at operational taxonomic unit (OTU) levels could be used to distinguish diseased soils from healthy soils (100), or OTU levels could be used to separate rice genotypes (99).

In our research, we discovered that RF model-based ASV levels were the most accurate at distinguishing between healthy and diseased rhizosphere microbial community signatures (Fig. 5). Furthermore, the model performed well at other taxonomic levels, such as when it was used at a family-level random forest classification model to distinguish indica and japonica root microbial communities (98). In conclusion, our results revealed that the diseased rhizosphere microbial community had consistent characteristics. This suggests that the rhizosphere microbial community can be used as a biomarker to diagnose disease in plants. Further in-depth research on marker bacteria can help to improve the accuracy of this diagnosis. This information can then be used to develop strategies to manage plant diseases.

### Conclusions

The alpha diversity of the BLB rhizobacterial community was reduced in this study, while the fungal community was not reduced. The bacterial community in the rhizosphere was more sensitive to BLB than the fungal community. The relative abundance of potentially beneficial microbes in the rhizosphere of BLB was higher. At the same time, the scale and complexity of the BLB rhizosphere bacterial community co-occurrence network increased, and the core species of the co-occurrence network comprised mainly beneficial bacteria. Furthermore, variations in BLB and soil nutrients especially pH and AP were connected to rhizosphere bacterial community and fungal community assembly. Taken together, these findings not only advance our understanding of microbial responses to environmental changes but also underscore the importance of harnessing the plant microbiome to promote plant health and support the development of sustainable agricultural ecosystems.

## Supplementary Material

Reviewer comments

## Data Availability

The data sets that support the findings of this study are available in the SRA at the NCBI with the identifier PRJNA806468.

## SUPPLEMENTAL MATERIAL

Supplemental material spectrum.01059-23-s0001.docx Fig. S1 to S9; Tables S1 to S5.

OPEN PEER REVIEW reviewer-comments.pdf An accounting of the reviewer comments and feedback.

ASM does not own the copyrights to Supplemental Material that may be linked to, or accessed through, an article. The authors have granted ASM a non-exclusive, world-wide license to publish the Supplemental Material files. Please contact the corresponding author directly for reuse.

## References

[1] Turner TR , James EK , Poole PS . 2013. The plant microbiome. Genome Biol 14:209. doi:10.1186/gb-2013-14-6-209 23805896 PMC3706808

[2] Bulgarelli D , Schlaeppi K , Spaepen S , Ver Loren van Themaat E , Schulze-Lefert P . 2013. Structure and functions of the bacterial microbiota of plants. Annu Rev Plant Biol 64:807–838. doi:10.1146/annurev-arplant-050312-120106 23373698

[3] Vandenkoornhuyse P , Quaiser A , Duhamel M , Le Van A , Dufresne A . 2015. The importance of the microbiome of the plant holobiont. New Phytol 206:1196–1206. doi:10.1111/nph.13312 25655016

[4] Trivedi P , Leach JE , Tringe SG , Sa T , Singh BK . 2020. Plant–microbiome interactions: from community assembly to plant health. Nat Rev Microbiol 18:607–621. doi:10.1038/s41579-020-0412-1 32788714

[5] Simon J-C , Marchesi JR , Mougel C , Selosse M-A . 2019. Host-microbiota interactions: from holobiont theory to analysis. Microbiome 7:5. doi:10.1186/s40168-019-0619-4 30635058 PMC6330386

[6] Kim YC , Leveau J , McSpadden Gardener BB , Pierson EA , Pierson LS , Ryu C-M . 2011. The multifactorial basis for plant health promotion by plant-associated bacteria. Appl Environ Microbiol 77:1548–1555. doi:10.1128/AEM.01867-10 21216911 PMC3067257

[7] Berg G . 2009. Plant–microbe interactions promoting plant growth and health: Perspectives for controlled use of microorganisms in agriculture. Appl Microbiol Biotechnol 84:11–18. doi:10.1007/s00253-009-2092-7 19568745

[8] Santhanam R , Luu VT , Weinhold A , Goldberg J , Oh Y , Baldwin IT . 2015. Native root-associated bacteria rescue a plant from a sudden-wilt disease that emerged during continuous cropping. Proc Natl Acad Sci U S A 112:E5013–20. doi:10.1073/pnas.1505765112 26305938 PMC4568709

[9] Pieterse CMJ , de Jonge R , Berendsen RL . 2016. The soil-borne supremacy. Trends Plant Sci 21:171–173. doi:10.1016/j.tplants.2016.01.018 26853594

[10] Miyauchi S , Kiss E , Kuo A , Drula E , Kohler A , Sánchez-García M , Morin E , Andreopoulos B , Barry KW , Bonito G , Buée M , Carver A , Chen C , Cichocki N , Clum A , Culley D , Crous PW , Fauchery L , Girlanda M , Hayes RD , Kéri Z , LaButti K , Lipzen A , Lombard V , Magnuson J , Maillard F , Murat C , Nolan M , Ohm RA , Pangilinan J , Pereira M de F , Perotto S , Peter M , Pfister S , Riley R , Sitrit Y , Stielow JB , Szöllősi G , Žifčáková L , Štursová M , Spatafora JW , Tedersoo L , Vaario L-M , Yamada A , Yan M , Wang P , Xu J , Bruns T , Baldrian P , Vilgalys R , Dunand C , Henrissat B , Grigoriev IV , Hibbett D , Nagy LG , Martin FM . 2020. Large-scale genome sequencing of mycorrhizal fungi provides insights into the early evolution of symbiotic traits. Nat Commun 11:5125. doi:10.1038/s41467-020-18795-w 33046698 PMC7550596

[11] Hacquard S , Garrido-Oter R , González A , Spaepen S , Ackermann G , Lebeis S , McHardy AC , Dangl JL , Knight R , Ley R , Schulze-Lefert P . 2015. Microbiota and host nutrition across plant and animal kingdoms. Cell Host Microbe 17:603–616. doi:10.1016/j.chom.2015.04.009 25974302

[12] Martin FM , Uroz S , Barker DG . 2017. Ancestral alliances: plant mutualistic symbioses with fungi and bacteria. Science 356:eaad4501. doi:10.1126/science.aad4501 28546156

[13] de Vries FT , Griffiths RI , Knight CG , Nicolitch O , Williams A . 2020. Harnessing rhizosphere microbiomes for drought-resilient crop production. Science 368:270–274. doi:10.1126/science.aaz5192 32299947

[14] Mendes R , Kruijt M , de Bruijn I , Dekkers E , van der Voort M , Schneider JHM , Piceno YM , DeSantis TZ , Andersen GL , Bakker P , Raaijmakers JM . 2011. Deciphering the rhizosphere microbiome for disease-suppressive bacteria. Science 332:1097–1100. doi:10.1126/science.1203980 21551032

[15] Carrión VJ , Perez-Jaramillo J , Cordovez V , Tracanna V , de Hollander M , Ruiz-Buck D , Mendes LW , van Ijcken WFJ , Gomez-Exposito R , Elsayed SS , Mohanraju P , Arifah A , van der Oost J , Paulson JN , Mendes R , van Wezel GP , Medema MH , Raaijmakers JM . 2019. Pathogen-induced activation of disease-suppressive functions in the endophytic root microbiome. Science 366:606–612. doi:10.1126/science.aaw9285 31672892

[16] Liu K , McInroy JA , Hu C-H , Kloepper JW . 2018. Mixtures of plant-growth-promoting rhizobacteria enhance biological control of multiple plant diseases and plant-growth promotion in the presence of pathogens. Plant Dis 102:67–72. doi:10.1094/PDIS-04-17-0478-RE 30673446

[17] Kandula DRW , Jones EE , Stewart A , McLean KL , Hampton JG . 2015. Trichoderma species for biocontrol of soil-borne plant pathogens of pasture species. Biocontrol Sci Technol 25:1052–1069. doi:10.1080/09583157.2015.1028892

[18] Ciancio A , Pieterse CMJ , Mercado-Blanco J . 2019. Editorial: harnessing useful rhizosphere microorganisms for pathogen and pest biocontrol - second edition. Front Microbiol 10:1935. doi:10.3389/fmicb.2019.01935 31555222 PMC6724568

[19] Bach E , Seger G dos S , Fernandes G de C , Lisboa BB , Passaglia LMP . 2016. Evaluation of biological control and rhizosphere competence of plant growth promoting bacteria. Applied Soil Ecology 99:141–149. doi:10.1016/j.apsoil.2015.11.002

[20] Edwards J , Johnson C , Santos-Medellín C , Lurie E , Podishetty NK , Bhatnagar S , Eisen JA , Sundaresan V . 2015. Structure, variation, and assembly of the root-associated Microbiomes of rice. Proc Natl Acad Sci U S A 112:E911–20. doi:10.1073/pnas.1414592112 25605935 PMC4345613

[21] Xiong C , Zhu Y-G , Wang J-T , Singh B , Han L-L , Shen J-P , Li P-P , Wang G-B , Wu C-F , Ge A-H , Zhang L-M , He J-Z . 2021. Host selection shapes crop microbiome assembly and network complexity. New Phytol 229:1091–1104. doi:10.1111/nph.16890 32852792

[22] Laforest-Lapointe I , Messier C , Kembel SW . 2016. Host species identity, site and time drive temperate tree phyllosphere bacterial community structure. Microbiome 4:27. doi:10.1186/s40168-016-0174-1 27316353 PMC4912770

[23] de Vries FT , Griffiths RI , Bailey M , Craig H , Girlanda M , Gweon HS , Hallin S , Kaisermann A , Keith AM , Kretzschmar M , Lemanceau P , Lumini E , Mason KE , Oliver A , Ostle N , Prosser JI , Thion C , Thomson B , Bardgett RD . 2018. Soil bacterial networks are less stable under drought than fungal networks. Nat Commun 9:3033. doi:10.1038/s41467-018-05516-7 30072764 PMC6072794

[24] Fernández-González AJ , Cardoni M , Gómez-Lama Cabanás C , Valverde-Corredor A , Villadas PJ , Fernández-López M , Mercado-Blanco J . 2020. Linking belowground microbial network changes to different tolerance level towards verticillium wilt of olive. Microbiome 8:11. doi:10.1186/s40168-020-0787-2 32007096 PMC6995654

[25] Shi W , Li M , Wei G , Tian R , Li C , Wang B , Lin R , Shi C , Chi X , Zhou B , Gao Z . 2019. The occurrence of potato common scab correlates with the community composition and function of the geocaulosphere soil microbiome. Microbiome 7:14. doi:10.1186/s40168-019-0629-2 30709420 PMC6359780

[26] Liu H , Li J , Carvalhais LC , Percy CD , Prakash Verma J , Schenk PM , Singh BK . 2021. Evidence for the plant recruitment of beneficial microbes to suppress soil-borne pathogens. New Phytol 229:2873–2885. doi:10.1111/nph.17057 33131088

[27] Yuan J , Zhao J , Wen T , Zhao M , Li R , Goossens P , Huang Q , Bai Y , Vivanco JM , Kowalchuk GA , Berendsen RL , Shen Q . 2018. Root exudates drive the soil-borne legacy of aboveground pathogen infection. Microbiome 6:156. doi:10.1186/s40168-018-0537-x 30208962 PMC6136170

[28] Lee S-M , Kong HG , Song GC , Ryu C-M . 2021. Disruption of firmicutes and actinobacteria abundance in tomato rhizosphere causes the incidence of bacterial wilt disease. ISME J 15:330–347. doi:10.1038/s41396-020-00785-x 33028974 PMC7852523

[29] Kwak M-J , Kong HG , Choi K , Kwon S-K , Song JY , Lee J , Lee PA , Choi SY , Seo M , Lee HJ , Jung EJ , Park H , Roy N , Kim H , Lee MM , Rubin EM , Lee S-W , Kim JF . 2018. Author correction: rhizosphere microbiome structure alters to enable wilt resistance in tomato. Nat Biotechnol 36:1117. doi:10.1038/nbt1118-1117 30412196

[30] Yang F , Zhang J , Zhang H , Ji G , Zeng L , Li Y , Yu C , Fernando WGD , Chen W . 2020. Bacterial blight induced shifts in endophytic microbiome of rice leaves and the enrichment of specific bacterial strains with pathogen antagonism. Front Plant Sci 11:963. doi:10.3389/fpls.2020.00963 32793250 PMC7390967

[31] Stefani E , Obradović A , Gašić K , Altin I , Nagy IK , Kovács T . 2021. Bacteriophage-mediated control of phytopathogenic xanthomonads: a promising green solution for the future. Microorganisms 9:1056. doi:10.3390/microorganisms9051056 34068401 PMC8153558

[32] Berendsen RL , Vismans G , Yu K , Song Y , de Jonge R , Burgman WP , Burmølle M , Herschend J , Bakker P , Pieterse CMJ . 2018. Disease-induced assemblage of a plant-beneficial bacterial consortium. ISME J 12:1496–1507. doi:10.1038/s41396-018-0093-1 29520025 PMC5956071

[33] Shekhar S , Kumar A . 2020. Field evaluation of different chemicals against bacterial leaf blight disease of rice caused by xanthomonas oryzae pv. oryzae. J Pharmacogn Phytochem 9:707–712.

[34] Zhang X , Liu S , Wang J , Huang Y , Freedman Z , Fu S , Liu K , Wang H , Li X , Yao M , Liu X , Schuler J . 2020. Local community assembly mechanisms shape soil bacterial Β diversity patterns along a latitudinal gradient. Nat Commun 11:5428. doi:10.1038/s41467-020-19228-4 33110057 PMC7591474

[35] Bolyen E , Rideout JR , Dillon MR , Bokulich NA , Abnet CC , Al-Ghalith GA , Alexander H , Alm EJ , Arumugam M , Asnicar F , Bai Y , Bisanz JE , Bittinger K , Brejnrod A , Brislawn CJ , Brown CT , Callahan BJ , Caraballo-Rodríguez AM , Chase J , Cope EK , Da Silva R , Diener C , Dorrestein PC , Douglas GM , Durall DM , Duvallet C , Edwardson CF , Ernst M , Estaki M , Fouquier J , Gauglitz JM , Gibbons SM , Gibson DL , Gonzalez A , Gorlick K , Guo J , Hillmann B , Holmes S , Holste H , Huttenhower C , Huttley GA , Janssen S , Jarmusch AK , Jiang L , Kaehler BD , Kang KB , Keefe CR , Keim P , Kelley ST , Knights D , Koester I , Kosciolek T , Kreps J , Langille MGI , Lee J , Ley R , Liu Y-X , Loftfield E , Lozupone C , Maher M , Marotz C , Martin BD , McDonald D , McIver LJ , Melnik AV , Metcalf JL , Morgan SC , Morton JT , Naimey AT , Navas-Molina JA , Nothias LF , Orchanian SB , Pearson T , Peoples SL , Petras D , Preuss ML , Pruesse E , Rasmussen LB , Rivers A , Robeson MS , Rosenthal P , Segata N , Shaffer M , Shiffer A , Sinha R , Song SJ , Spear JR , Swafford AD , Thompson LR , Torres PJ , Trinh P , Tripathi A , Turnbaugh PJ , Ul-Hasan S , van der Hooft JJJ , Vargas F , Vázquez-Baeza Y , Vogtmann E , von Hippel M , Walters W , Wan Y , Wang M , Warren J , Weber KC , Williamson CHD , Willis AD , Xu ZZ , Zaneveld JR , Zhang Y , Zhu Q , Knight R , Caporaso JG . 2019. Reproducible, interactive, scalable and extensible microbiome data science using QIIME 2. Nat Biotechnol 37:852–857. doi:10.1038/s41587-019-0252-6 31341288 PMC7015180

[36] Martin M . 2011. Cutadapt removes adapter sequences from high-throughput sequencing reads. EMBnet j 17:10. doi:10.14806/ej.17.1.200

[37] Callahan BJ , McMurdie PJ , Rosen MJ , Han AW , Johnson AJA , Holmes SP . 2016. DADA2: high-resolution sample inference from Illumina amplicon data. Nat Methods 13:581–583. doi:10.1038/nmeth.3869 27214047 PMC4927377

[38] Bokulich NA , Kaehler BD , Rideout JR , Dillon M , Bolyen E , Knight R , Huttley GA , Gregory Caporaso J . 2018. Optimizing taxonomic classification of marker-gene amplicon sequences with QIIME 2’s q2-feature-classifier plugin. Microbiome 6:90. doi:10.1186/s40168-018-0470-z 29773078 PMC5956843

[39] Bolger AM , Lohse M , Usadel B . 2014. Trimmomatic: a flexible trimmer for Illumina sequence data. Bioinformatics 30:2114–2120. doi:10.1093/bioinformatics/btu170 24695404 PMC4103590

[40] Wood DE , Salzberg SL . 2014. Kraken: ultrafast metagenomic sequence classification using exact alignments. Genome Biol 15:R46. doi:10.1186/gb-2014-15-3-r46 24580807 PMC4053813

[41] Li D , Liu C-M , Luo R , Sadakane K , Lam T-W . 2015. MEGAHIT: an ultra-fast single-node solution for large and complex metagenomics assembly via succinct de bruijn graph. Bioinformatics 31:1674–1676. doi:10.1093/bioinformatics/btv033 25609793

[42] Hyatt D , Chen G-L , Locascio PF , Land ML , Larimer FW , Hauser LJ . 2010. Prodigal: prokaryotic gene recognition and translation initiation site identification. BMC Bioinformatics 11:119. doi:10.1186/1471-2105-11-119 20211023 PMC2848648

[43] Fu L , Niu B , Zhu Z , Wu S , Li W . 2012. CD-HIT: accelerated for clustering the next-generation sequencing data. Bioinformatics 28:3150–3152. doi:10.1093/bioinformatics/bts565 23060610 PMC3516142

[44] Segata N , Izard J , Waldron L , Gevers D , Miropolsky L , Garrett WS , Huttenhower C . 2011. Metagenomic biomarker discovery and explanation. Genome Biol 12:R60. doi:10.1186/gb-2011-12-6-r60 21702898 PMC3218848

[45] Lombard V , Golaconda Ramulu H , Drula E , Coutinho PM , Henrissat B . 2014. The carbohydrate-active enzymes database (CAZy) in 2013. Nucleic Acids Res 42:D490–5. doi:10.1093/nar/gkt1178 24270786 PMC3965031

[46] Huerta-Cepas J , Szklarczyk D , Forslund K , Cook H , Heller D , Walter MC , Rattei T , Mende DR , Sunagawa S , Kuhn M , Jensen LJ , von Mering C , Bork P . 2016. eggNOG 4.5: a hierarchical orthology framework with improved functional annotations for eukaryotic, prokaryotic and viral sequences. Nucleic Acids Res 44:D286–93. doi:10.1093/nar/gkv1248 26582926 PMC4702882

[47] Xiong C , Singh BK , He J-Z , Han Y-L , Li P-P , Wan L-H , Meng G-Z , Liu S-Y , Wang J-T , Wu C-F , Ge A-H , Zhang L-M . 2021. Plant developmental stage drives the differentiation in ecological role of the maize microbiome. Microbiome 9:171. doi:10.1186/s40168-021-01118-6 34389047 PMC8364065

[48] Bastian M , Heymann S , Jacomy M . n.d. Gephi: An open source software for exploring and manipulating networks 3:361–362. doi:10.1609/icwsm.v3i1.13937

[49] McArdle BH , Anderson MJ . 2001. Fitting multivariate models to community data: a comment on distance‐based redundancy analysis. Ecology 82:290–297. doi:10.1890/0012-9658(2001)082[0290:FMMTCD]2.0.CO;2

[50] Sadeghi A , Karimi E , Dahaji PA , Javid MG , Dalvand Y , Askari H . 2012. Plant growth promoting activity of an auxin and siderophore producing isolate of streptomyces under saline soil conditions. World J Microbiol Biotechnol 28:1503–1509. doi:10.1007/s11274-011-0952-7 22805932

[51] Philippot L , Raaijmakers JM , Lemanceau P , van der Putten WH . 2013. Going back to the roots: the microbial ecology of the rhizosphere. Nat Rev Microbiol 11:789–799. doi:10.1038/nrmicro3109 24056930

[52] Zhalnina K , Louie KB , Hao Z , Mansoori N , da Rocha UN , Shi S , Cho H , Karaoz U , Loqué D , Bowen BP , Firestone MK , Northen TR , Brodie EL . 2018. Dynamic root exudate chemistry and microbial substrate preferences drive patterns in rhizosphere microbial community assembly. Nat Microbiol 3:470–480. doi:10.1038/s41564-018-0129-3 29556109

[53] Mendes R , Garbeva P , Raaijmakers JM . 2013. The rhizosphere microbiome: significance of plant beneficial, plant pathogenic, and human pathogenic microorganisms. FEMS Microbiol Rev 37:634–663. doi:10.1111/1574-6976.12028 23790204

[54] Bakker P , Berendsen RL , Doornbos RF , Wintermans PCA , Pieterse CMJ . 2013. The rhizosphere revisited: root microbiomics. Front Plant Sci 4:165. doi:10.3389/fpls.2013.00165 23755059 PMC3667247

[55] Gómez Expósito R , de Bruijn I , Postma J , Raaijmakers JM . 2017. Current insights into the role of Rhizosphere bacteria in disease suppressive soils. Front. Microbiol 8:2529. doi:10.3389/fmicb.2017.02529 29326674 PMC5741648

[56] Sun X , Xu Z , Xie J , Hesselberg-Thomsen V , Tan T , Zheng D , Strube ML , Dragoš A , Shen Q , Zhang R , Kovács ÁT . 2022. Bacillus velezensis stimulates resident rhizosphere Pseudomonas stutzeri for plant health through metabolic interactions. ISME J 16:774–787. doi:10.1038/s41396-021-01125-3 34593997 PMC8483172

[57] Wei F , Feng H , Zhang D , Feng Z , Zhao L , Zhang Y , Deakin G , Peng J , Zhu H , Xu X . 2021. Composition of rhizosphere microbial communities associated with healthy and Verticillium wilt diseased cotton plants. Front Microbiol 12:618169. doi:10.3389/fmicb.2021.618169 33889135 PMC8057349

[58] Gao M , Xiong C , Gao C , Tsui CKM , Wang M-M , Zhou X , Zhang A-M , Cai L . 2021. Disease-induced changes in plant microbiome assembly and functional adaptation. Microbiome 9:187. doi:10.1186/s40168-021-01138-2 34526096 PMC8444440

[59] Faust K , Raes J . 2012. Microbial interactions: from networks to models. Nat Rev Microbiol 10:538–550. doi:10.1038/nrmicro2832 22796884

[60] Coyte KZ , Schluter J , Foster KR . 2015. The ecology of the microbiome: networks, competition, and stability. Science 350:663–666. doi:10.1126/science.aad2602 26542567

[61] Grilli J , Rogers T , Allesina S . 2016. Modularity and stability in ecological communities. Nat Commun 7:12031. doi:10.1038/ncomms12031 27337386 PMC4931019

[62] Berendsen RL , Pieterse CMJ , Bakker P . 2012. The rhizosphere microbiome and plant health. Trends Plant Sci 17:478–486. doi:10.1016/j.tplants.2012.04.001 22564542

[63] Cha J-Y , Han S , Hong H-J , Cho H , Kim D , Kwon Y , Kwon S-K , Crüsemann M , Bok Lee Y , Kim JF , Giaever G , Nislow C , Moore BS , Thomashow LS , Weller DM , Kwak Y-S . 2016. Microbial and biochemical basis of a fusarium wilt-suppressive soil. ISME J 10:119–129. doi:10.1038/ismej.2015.95 26057845 PMC4681868

[64] Schulz-Bohm K , Gerards S , Hundscheid M , Melenhorst J , de Boer W , Garbeva P . 2018. Calling from distance: attraction of soil bacteria by plant root volatiles. ISME J 12:1252–1262. doi:10.1038/s41396-017-0035-3 29358736 PMC5931972

[65] Olanrewaju OS , Babalola OO . 2019. Streptomyces: implications and interactions in plant growth promotion. Appl Microbiol Biotechnol 103:1179–1188. doi:10.1007/s00253-018-09577-y 30594952 PMC6394478

[66] Li P-D , Zhu Z-R , Zhang Y , Xu J , Wang H , Wang Z , Li H . 2022. The phyllosphere microbiome shifts toward combating melanose pathogen. Microbiome 10:56. doi:10.1186/s40168-022-01234-x 35366955 PMC8976405

[67] Matsumoto H , Fan X , Wang Y , Kusstatscher P , Duan J , Wu S , Chen S , Qiao K , Wang Y , Ma B , Zhu G , Hashidoko Y , Berg G , Cernava T , Wang M . 2021. Bacterial seed endophyte shapes disease resistance in rice. Nat Plants 7:60–72. doi:10.1038/s41477-020-00826-5 33398157

[68] Caulier S , Nannan C , Gillis A , Licciardi F , Bragard C , Mahillon J . 2019. Overview of the antimicrobial compounds produced by members of the Bacillus subtilis group. Front Microbiol 10:302. doi:10.3389/fmicb.2019.00302 30873135 PMC6401651

[69] Álvarez-Pérez JM , González-García S , Cobos R , Olego MÁ , Ibañez A , Díez-Galán A , Garzón-Jimeno E , Coque JJR . 2017. Use of endophytic and rhizosphere actinobacteria from grapevine plants to reduce nursery fungal graft infections that lead to young grapevine decline. Appl Environ Microbiol 83:e01564-17. doi:10.1128/AEM.01564-17 28986378 PMC5717199

[70] Verma VC , Singh SK , Prakash S . 2011. Bio-control and plant growth promotion potential of siderophore producing endophytic streptomyces from azadirachta indica A. Juss. J Basic Microbiol 51:550–556. doi:10.1002/jobm.201000155 21656792

[71] Poudel R , Jumpponen A , Schlatter DC , Paulitz TC , Gardener BBM , Kinkel LL , Garrett KA . 2016. Microbiome networks: a systems framework for identifying candidate microbial assemblages for disease management. Phytopathology 106:1083–1096. doi:10.1094/PHYTO-02-16-0058-FI 27482625

[72] Toju H , Peay KG , Yamamichi M , Narisawa K , Hiruma K , Naito K , Fukuda S , Ushio M , Nakaoka S , Onoda Y , Yoshida K , Schlaeppi K , Bai Y , Sugiura R , Ichihashi Y , Minamisawa K , Kiers ET . 2018. Core microbiomes for sustainable agroecosystems. Nat Plants 4:733. doi:10.1038/s41477-018-0245-3 30108297

[73] Banerjee S , Schlaeppi K , van der Heijden MGA . 2018. Keystone taxa as drivers of microbiome structure and functioning. Nat Rev Microbiol 16:567–576. doi:10.1038/s41579-018-0024-1 29789680

[74] Durán P , Thiergart T , Garrido-Oter R , Agler M , Kemen E , Schulze-Lefert P , Hacquard S . 2018. Microbial interkingdom interactions in roots promote arabidopsis survival. Cell 175:973–983. doi:10.1016/j.cell.2018.10.020 30388454 PMC6218654

[75] New FN , Brito IL . 2020. What is metagenomics teaching us, and what is missed? Annu Rev Microbiol 74:117–135. doi:10.1146/annurev-micro-012520-072314 32603623

[76] Quince C , Walker AW , Simpson JT , Loman NJ , Segata N . 2017. Corrigendum: shotgun metagenomics, from sampling to analysis. Nat Biotechnol 35:1211. doi:10.1038/nbt1217-1211b 29220029

[77] Delgado-Baquerizo M , Maestre FT , Reich PB , Jeffries TC , Gaitan JJ , Encinar D , Berdugo M , Campbell CD , Singh BK . 2016. Microbial diversity drives multifunctionality in terrestrial ecosystems. Nat Commun 7:10541. doi:10.1038/ncomms10541 26817514 PMC4738359

[78] Isbell F , Calcagno V , Hector A , Connolly J , Harpole WS , Reich PB , Scherer-Lorenzen M , Schmid B , Tilman D , van Ruijven J , Weigelt A , Wilsey BJ , Zavaleta ES , Loreau M . 2011. High plant diversity is needed to maintain ecosystem services. Nature 477:199–202. doi:10.1038/nature10282 21832994

[79] Hector A , Bagchi R . 2007. Biodiversity and ecosystem multifunctionality. Nature 448:188–190. doi:10.1038/nature05947 17625564

[80] Wagg C , Bender SF , Widmer F , van der Heijden MGA . 2014. Soil biodiversity and soil community composition determine ecosystem multifunctionality. Proc Natl Acad Sci U S A 111:5266–5270. doi:10.1073/pnas.1320054111 24639507 PMC3986181

[81] Wagg C , Schlaeppi K , Banerjee S , Kuramae EE , van der Heijden MGA . 2019. Fungal-bacterial diversity and microbiome complexity predict ecosystem functioning. Nat Commun 10:4841. doi:10.1038/s41467-019-12798-y 31649246 PMC6813331

[82] Deutscher J , Francke C , Postma PW . 2006. How phosphotransferase system-related protein phosphorylation regulates carbohydrate metabolism in bacteria. Microbiol Mol Biol Rev 70:939–1031. doi:10.1128/MMBR.00024-06 17158705 PMC1698508

[83] Patipong T , Ngoennet S , Honda M , Hibino T , Waditee-Sirisattha R , Kageyama H . 2019. A class I fructose-1,6-bisphosphate aldolase is associated with salt stress tolerance in a halotolerant cyanobacterium halothece sp. PCC 7418. Arch Biochem Biophys 672:108059. doi:10.1016/j.abb.2019.07.024 31356779

[84] Pandey A , Sonti RV . 2010. Role of the feob protein and siderophore in promoting virulence of xanthomonas oryzae pv. oryzae on rice. J Bacteriol 192:3187–3203. doi:10.1128/JB.01558-09 20382771 PMC2901680

[85] Vikram A , Bomberger JM , Bibby KJ . 2015. Efflux as a glutaraldehyde resistance mechanism in pseudomonas fluorescens and Pseudomonas aeruginosa biofilms. Antimicrob Agents Chemother 59:3433–3440. doi:10.1128/AAC.05152-14 25824217 PMC4432172

[86] Prell J , White JP , Bourdes A , Bunnewell S , Bongaerts RJ , Poole PS . 2009. Legumes regulate rhizobium bacteroid development and persistence by the supply of branched-chain amino acids. Proc Natl Acad Sci U S A 106:12477–12482. doi:10.1073/pnas.0903653106 19597156 PMC2718340

[87] Ragot SA , Kertesz MA , Bünemann EK . 2015. phoD alkaline phosphatase gene diversity in soil. Appl Environ Microbiol 81:7281–7289. doi:10.1128/AEM.01823-15 26253682 PMC4579420

[88] Conrad R . 2009. The global methane cycle: recent advances in understanding the microbial processes involved. Environ Microbiol Rep 1:285–292. doi:10.1111/j.1758-2229.2009.00038.x 23765881

[89] Treu L , Campanaro S , Kougias PG , Zhu X , Angelidaki I . 2016. Untangling the effect of fatty acid addition at species level revealed different transcriptional responses of the biogas microbial community members. Environ Sci Technol 50:6079–6090. doi:10.1021/acs.est.6b00296 27154312

[90] Li A , Chu Y , Wang X , Ren L , Yu J , Liu X , Yan J , Zhang L , Wu S , Li S . 2013. A pyrosequencing-based metagenomic study of methane-producing microbial community in solid-state biogas reactor. Biotechnol Biofuels 6:3. doi:10.1186/1754-6834-6-3 23320936 PMC3618299

[91] Wells M , Kanmanii NJ , Al Zadjali AM , Janecka JE , Basu P , Oremland RS , Stolz JF . 2020. Methane, arsenic, selenium and the origins of the DMSO reductase family. Sci Rep 10:10946. doi:10.1038/s41598-020-67892-9 32616801 PMC7331816

[92] Adam PS , Borrel G , Gribaldo S . 2018. Evolutionary history of carbon monoxide dehydrogenase/acetyl-CoA synthase, one of the oldest enzymatic complexes. Biotechnol Biofuels 115:E1166–E1173. doi:10.1073/pnas.1716667115 PMC5819426 29358391

[93] Nemergut DR , Schmidt SK , Fukami T , O’Neill SP , Bilinski TM , Stanish LF , Knelman JE , Darcy JL , Lynch RC , Wickey P , Ferrenberg S . 2013. Patterns and processes of microbial community assembly. Microbiol Mol Biol Rev 77:342–356. doi:10.1128/MMBR.00051-12 24006468 PMC3811611

[94] Ramoneda J , Le Roux JJ , Frossard E , Frey B , Gamper HA . 2020. Experimental assembly reveals ecological drift as a major driver of root nodule bacterial diversity in a woody legume crop. FEMS Microbiol Ecol 96:fiaa083. doi:10.1093/femsec/fiaa083 32364226

[95] Fillinger L , Hug K , Griebler C . 2019. Selection imposed by local environmental conditions drives differences in microbial community composition across geographically distinct groundwater aquifers. FEMS Microbiol Ecol 95:fiz160. doi:10.1093/femsec/fiz160 31598689 PMC6821248

[96] Luan L , Liang C , Chen L , Wang H , Xu Q , Jiang Y , Sun B . 2020. Coupling bacterial community assembly to microbial metabolism across soil profiles. mSystems 5:e00298-20. doi:10.1128/mSystems.00298-20 32518195 PMC7289589

[97] Wen T , Xie P , Penton CR , Hale L , Thomashow LS , Yang S , Ding Z , Su Y , Yuan J , Shen Q . 2022. Specific metabolites drive the deterministic assembly of diseased rhizosphere microbiome through weakening microbial degradation of autotoxin. Microbiome 10:177. doi:10.1186/s40168-022-01375-z 36271396 PMC9587672

[98] Zhang J , Liu Y-X , Zhang N , Hu B , Jin T , Xu H , Qin Y , Yan P , Zhang X , Guo X , Hui J , Cao S , Wang X , Wang C , Wang H , Qu B , Fan G , Yuan L , Garrido-Oter R , Chu C , Bai Y . 2019. NRT1. 1B is associated with root microbiota composition and nitrogen use in field-grown rice. Nat Biotechnol 37:676–684. doi:10.1038/s41587-019-0104-4 31036930

[99] Xiong J , Lu J , Li X , Qiu Q , Chen J , Yan C . 2021. Effect of rice (Oryza sativa L.) genotype on yield: evidence from recruiting spatially consistent rhizosphere microbiome. Soil Biology and Biochemistry 161:108395. doi:10.1016/j.soilbio.2021.108395

[100] Yuan J , Wen T , Zhang H , Zhao M , Penton CR , Thomashow LS , Shen Q . 2020. Predicting disease occurrence with high accuracy based on soil macroecological patterns of fusarium wilt. ISME J 14:2936–2950. doi:10.1038/s41396-020-0720-5 32681158 PMC7784920

