# Supplementary material for: Rice bacterial leaf blight drives rhizosphere microbial assembly and function adaptation
Source: Microbiol Spectr. 2023 Oct 17;11(6):e01059-23. doi: 10.1128/spectrum.01059-23 (PMC10715139; doi:10.1128/spectrum.01059-23)
Supplement: Supplemental material — Fig. S1 to S9; Tables S1 to S5. [file spectrum.01059-23-s0001.docx]

**Rice bacterial leaf blight drive rhizosphere microbial assembly and function adaptation**

Hubiao jiang^a^, Jinyan Luo^b^, Quanhong Liu^a^, Solabomi Olaitan Ogunyemi^a^, Temoor Ahmed^a^, Bing Li^a^, Shanhong Yu^c^, Xiao Wang^d^, Chenqi Yan^e^, Jianping Chen^f,^*, Bin Li^a,^*

^a^State Key Laboratory of Rice Biology, Ministry of Agriculture Key Laboratory of Molecular Biology of Crop Pathogens and Insects, Key Laboratory of Biology of Crop Pathogens and Insects of Zhejiang Province, Institute of Biotechnology, Zhejiang University, Hangzhou 310058, China.

^b^Department of Plant Quarantine, Shanghai Extension and Service Center of Agriculture Technology, Shanghai 201103, China.

^c^Taizhou Academy of Agricultural Sciences, Taizhou 317000, China

^d^Ningbo Jiangbei District Agricultural Technology Extension Service Station, Ningbo 315033, China

^e^Institute of Biotechnology, Ningbo Academy of Agricultural Sciences, Ningbo 315040, China

^f^State Key Laboratory for Managing Biotic and Chemical Threats to the Quality and Safety of Agro-products, Key Laboratory of Biotechnology in Plant Protection of Ministry of Agriculture and Zhejiang Province, Institute of Plant Virology, Ningbo University, Ningbo, 315211, China

* Correspondence: jpchen2001@126.com (J.C.); libin0571@zju.edu.cn (B.L.); +86 0571-88982412.

**
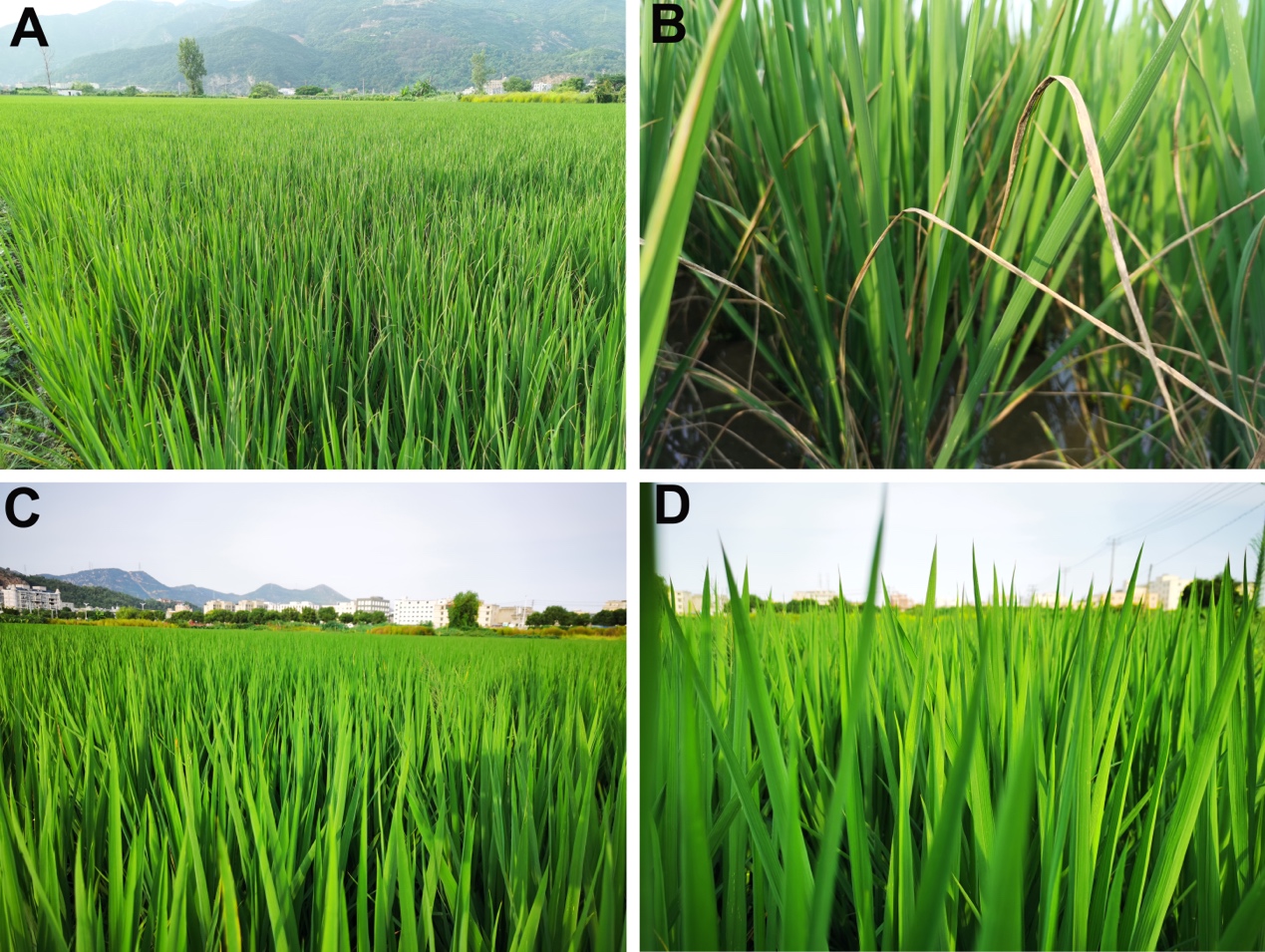
**

**Fig. S1** Rice bacterial blight symptoms. A and B are diseased rice, C and D are healthy rice**.**

**
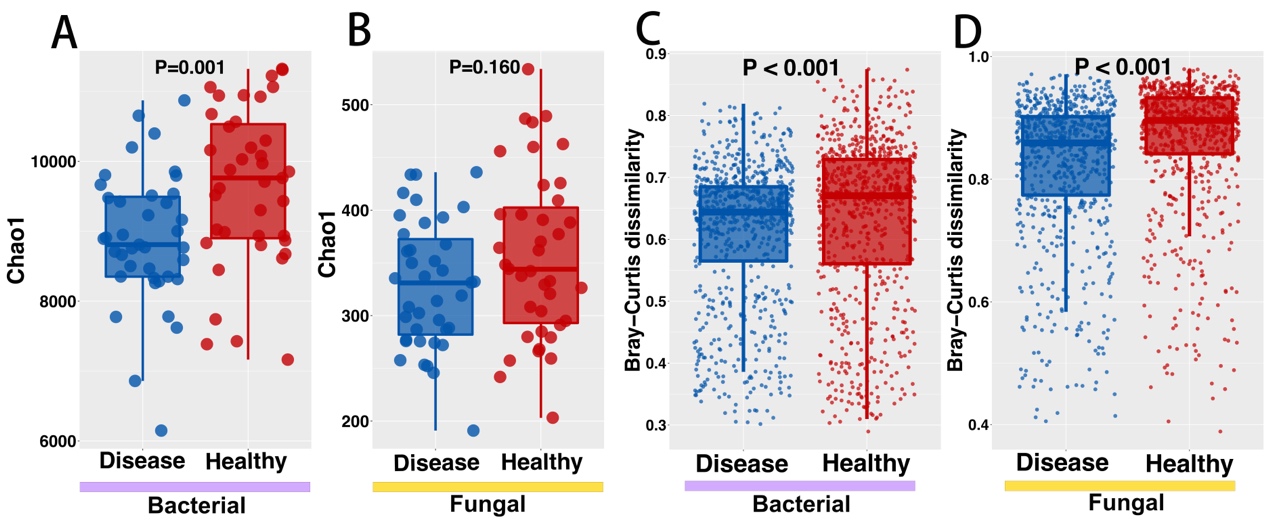
**

**Fig. S2** Overall alpha and beta diversity of the rhizosphere microbial community in healthy and diseased group. Bacterial (A) and fungal (B) alpha diversity. Bacteria (C) and fungal (D) based on the beta dissimilarity of Bray_curtis distance matrix.

**
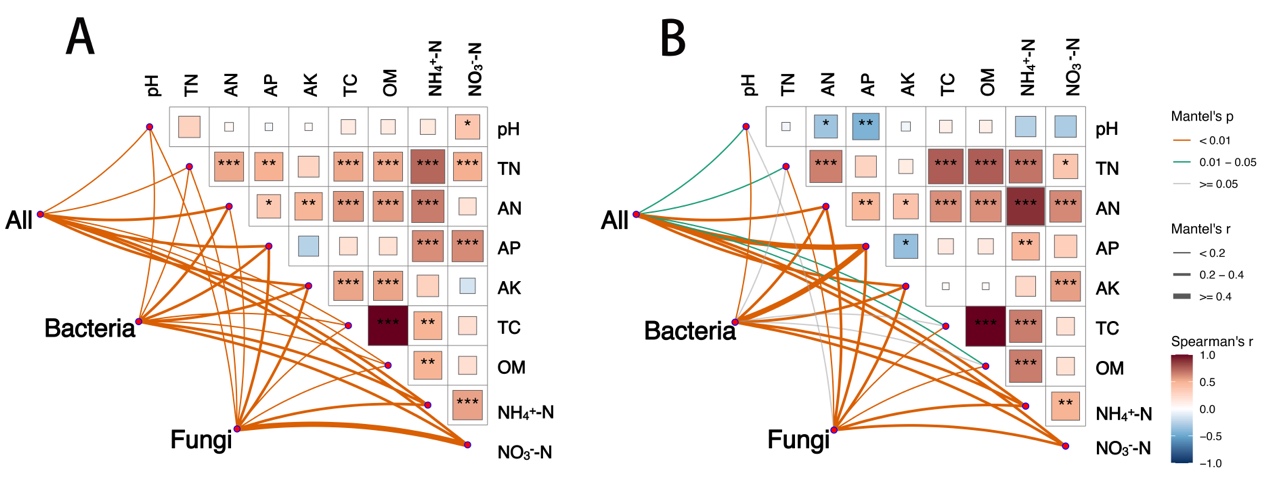
**

**Fig. S3** Correlations between environmental factors and rhizosphere-related taxa. Spearman correlations between diseased (A) and healthy (B) rhizosphere bacterial and fungal communities and soil physicochemical properties. All, the entire bacterial and fungal community; Bacteria, the entire bacterial community; Fungi, the entire fungal community. Edge widths correspond to Mantel's R statistic and edge colors indicate statistical significance based on 999 permutations. pH, soil pH; AP, available phosphorus; AN, available nitrogen; AK, available potassium; NH_4_^+^-N, ammonium; NO_3_^−^-N, nitrate; TC, total carbon; OM, organic matter; TN, total nitrogen. Asterisks indicate significant differences (*: *P* < 0.05; **: *P* < 0.01; ***: *P* < 0.001).

**
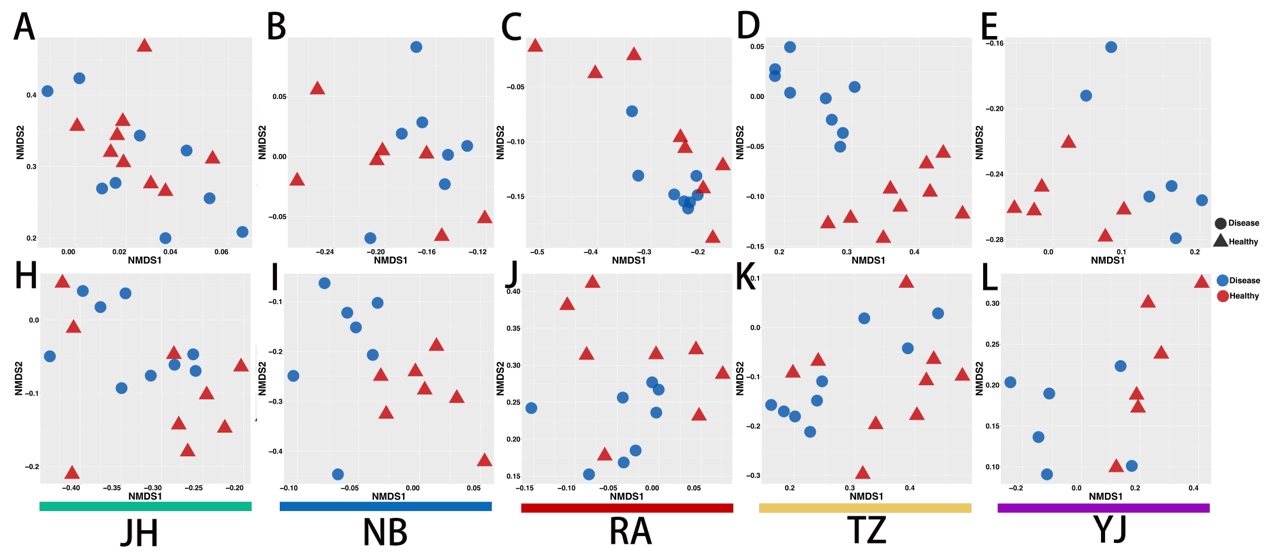
**

**Fig. S4** NMDS (Non-Metric Multidimensional Scaling) based Bray-Curtis matrix beta diversity analysis of bacteria (A-E) and fungi (H-L). Significant differences in communities were tested using PERMANOVA. NB: Ningbo samples, RA: Ruian samples, YJ: Yongjia samples, JH: Jinhua samples, TZ: Taizhou samples.

**
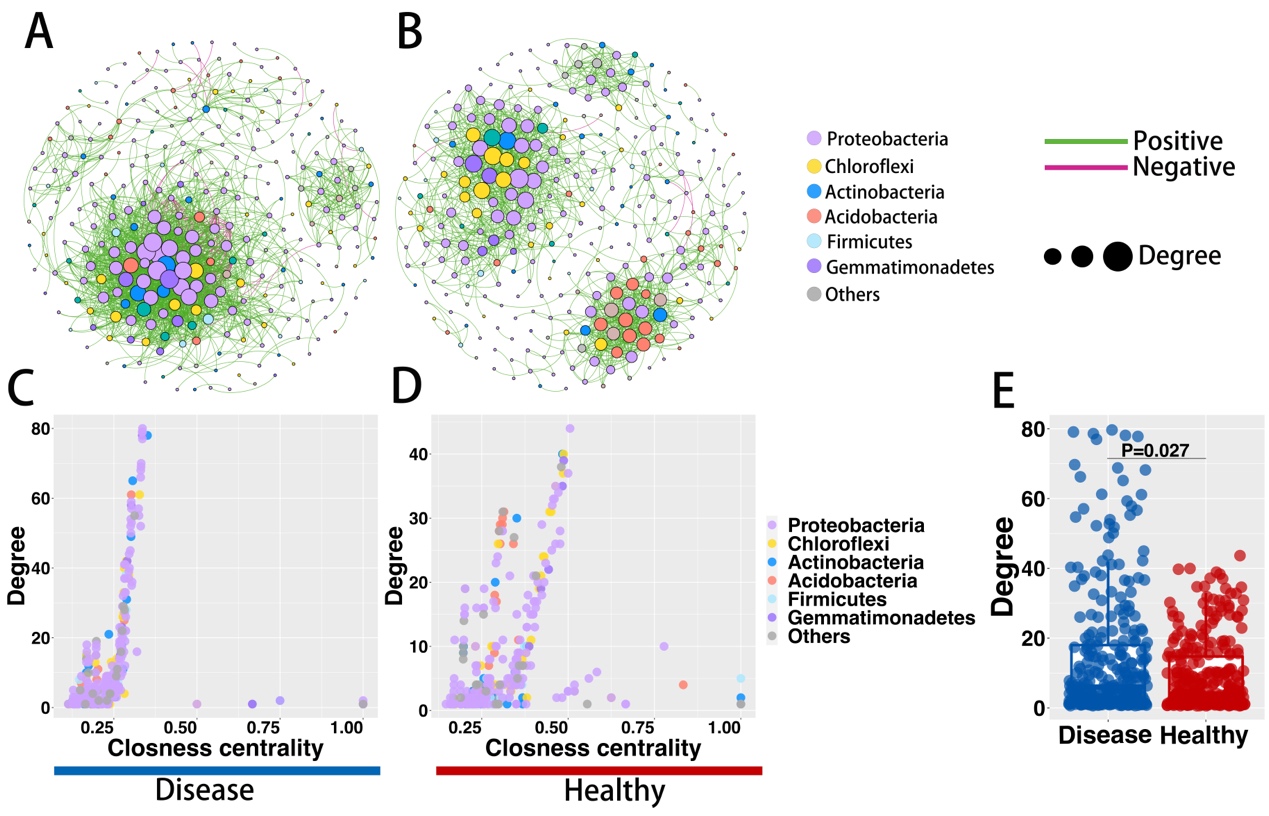
**

**Fig. S5** Co-occurrence networks bacterial in diseased (A) and healthy (B) rhizospheres. Diseased (C) and d Healthy (D)comparison of networks node-level topological features (degree and closeness centrality). (E) Degree values of bacterial and fungal taxa in healthy and diseased networks. The significance of difference was determined by nonparametric Kruskal–Wallis test. Node size indicates the network degree. The nodes were colored according to bacterial phylum. Correlations are indicated between nodes (correlation coefficient > 0.7 indicates positive correlation, the green line indicates positive correlation; correlation coefficient < -0.7 indicates a negative correlation, and the red line indicates negative correlation).

**
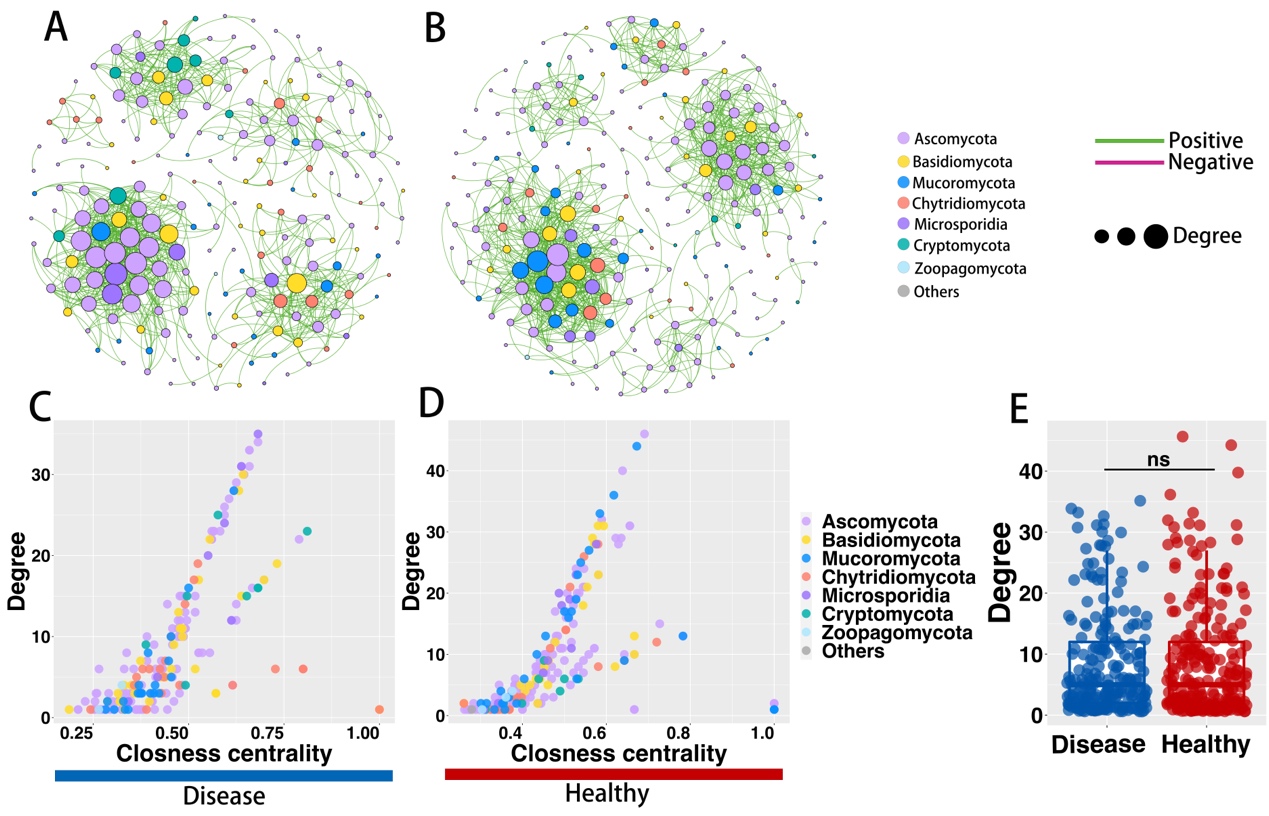
**

**Fig. S6** Co-occurrence networks fungal in diseased (A) and healthy (B) rhizospheres. Diseased (C) and d Healthy (D) comparison of networks node-level topological features (degree and closeness centrality). (E) Degree values of bacterial and fungal taxa in healthy and diseased networks. The significance of difference was determined by nonparametric Kruskal–Wallis test. Node size indicates the network degree. The nodes were colored according to fungal phylum. Correlations are indicated between nodes (correlation coefficient > 0.7 indicates positive correlation, the green line indicates positive correlation; correlation coefficient < -0.7 indicates a negative correlation, and the red line indicates negative correlation).

**
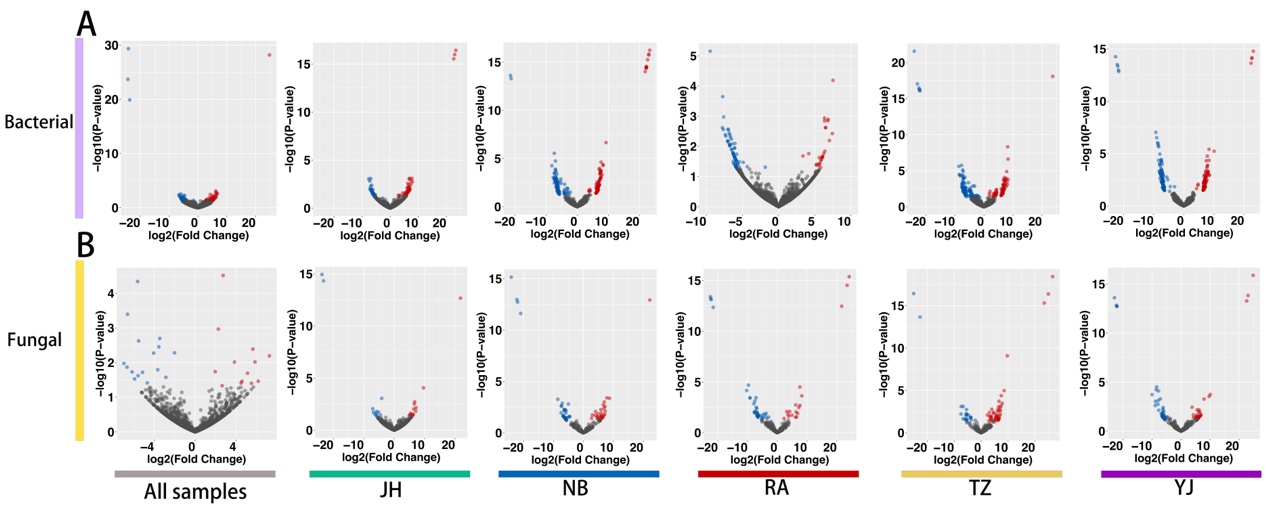
**

**Fig. S7** Volcano plots showing enrichment and depletion patterns of bacterial (A) and fungal (B) microbial communities in diseased rhizosphere compared to healthy rhizosphere. Red dots represent enriched ASVs, blue for depleted ASVs, and gray for none difference ASV. NB: Ningbo samples, RA: Ruian samples, YJ: Yongjia samples, JH: Jinhua samples, TZ: Taizhou samples.


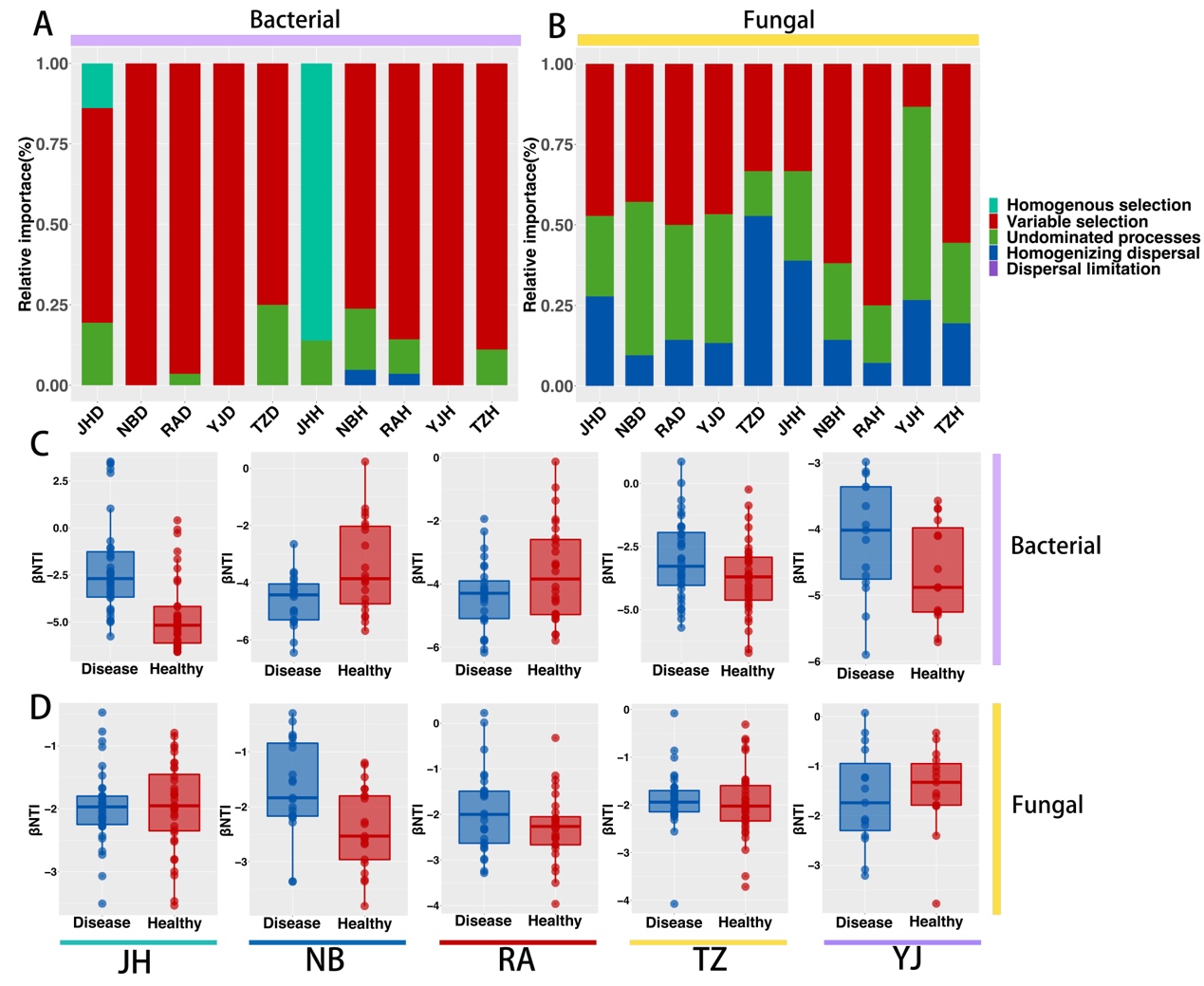


**Fig. S8** Assembly process of healthy and diseased rhizosphere microbial communities (bacteria and fungi). The relative importance of bacterial (A) and fungal (B) community assembly processes. The weighted β nearest taxon index (βNTI) for bacterial (C) and fungal (D) community assembly.

**
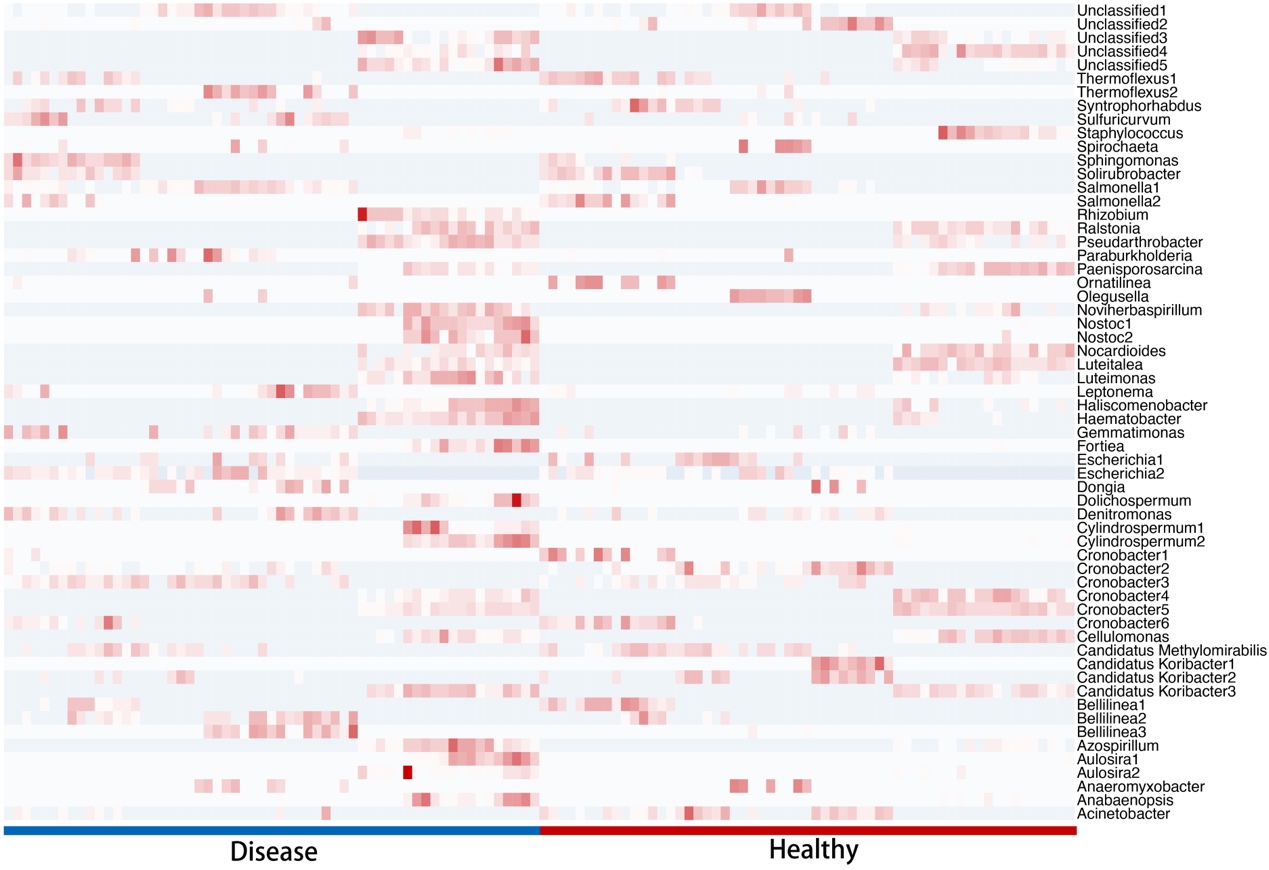
**

**Fig. S9** Heatmaps of the relative abundance of the top 60 biomarkers in the healthy and diseased rhizosphere detected by a random forest model that accurately predict health and disease.

**Table S1.** Significance test of beta diversity estimates between all samples.

| **Explanatory factors** | **R2** | **P** | **group** | **Method** |
| --- | --- | --- | --- | --- |
| Sampling site | 0.543 | 0.001 | All bacterial samples | PERMANOVA |
| BLB | 0.015 | 0.01 | All bacterial samples | PERMANOVA |
| Sampling site | 0.381 | 0.001 | All fungal samples | PERMANOVA |
| BLB | 0.023 | 0.001 | All fungal samples | PERMANOVA |
| BLB | 0.08 | 0.041 | JH bacterial samples | PERMANOVA |
| BLB | 0.104 | 0.049 | JH fungal samples | PERMANOVA |
| BLB | 0.159 | 0.001 | NB bacterial samples | PERMANOVA |
| BLB | 0.162 | 0.003 | NB fungal samples | PERMANOVA |
| BLB | 0.098 | 0.045 | RA bacterial samples | PERMANOVA |
| BLB | 0.164 | 0.002 | RA fungal samples | PERMANOVA |
| BLB | 0.186 | 0.001 | TZ bacterial samples | PERMANOVA |
| BLB | 0.137 | 0.002 | TZ fungal samples | PERMANOVA |
| BLB | 0.225 | 0.005 | YJ bacterial samples | PERMANOVA |
| BLB | 0.188 | 0.004 | YJ fungal samples | PERMANOVA |
| BLB | 0.586 | 0.037 | TZ KO samples | PERMANOVA |
| BLB | 0.693 | 0.022 | TZ CAZy samples | PERMANOVA |
| BLB | 0.684 | 0.022 | TZ COG samples | PERMANOVA |

**Table S2. Topological properties of microbial networks.**

| **Topological properties** | **All samples** | | **All bacterial samples** | | **All fungal samples** | |
| --- | --- | --- | --- | --- | --- | --- |
|  | **Disease** | **Healthy** | **Disease** | **Healthy** | **Disease** | **Healthy** |
| **Average degree^a^** | 13.371 | 10.072 | 13.935 | 9.329 | 8.461 | 8.79 |
| **Average weighted degree^b^** | 11.346 | 8.542 | 11.853 | 7.917 | 7.284 | 7.544 |
| **Network diameter^c^** | 13 | 15 | 15 | 11 | 8 | 6 |
| **Modularity^d^** | 0.449 | 0.661 | 0.357 | 0.63 | 0.678 | 0.656 |
| **Clustering coefficient^e^** | 0.588 | 0.575 | 0.586 | 0.596 | 0.649 | 0.652 |
| **Average path length^f^** | 4.508 | 6.133 | 4.699 | 3.572 | 2.508 | 2.313 |
| **Number of edges^g^** | 2507 | 1813 | 2139 | 1390 | 918 | 1046 |
| **Number of nodes^h^** | 375 | 360 | 307 | 298 | 217 | 238 |
| **Negative edges^i^** | 85 | 9 | 78 | 5 | 0 | 0 |
| **Positive edges^j^** | 2422 | 1804 | 2061 | 1385 | 918 | 1046 |
| **Closness centrality^k^** | 0.279 | 0.253 | 0.278 | 0.361 | 0.466 | 0.501 |
| **Betweeness centrality^l^** | 524.859 | 672.242 | 484.391 | 143.953 | 36.120 | 31.992 |

a: The node connectivity, that is, the average number of connections per node in the network.

b: The average of the weighted degrees of all nodes in the network.

c:The longest distance between nodes in the network, measured in number of edges.

d:The ability of the nodes to form highly associated communities, that is, the structure with more complicated between nodes connections.

e:The degree that the nodes tend to cluster together, and how nodes are embedded in the community.

f:Average network distance between all pair of nodes or the average length off all edges in the network.

g: The number of connections obtained by spearman correlations.

h:Microbial taxon (at ASV level) with at least one significant (P < 0.05) and strong (spearman > 0.7 or < -0.7) correlation.

i: spearman negative correlation (< -0.7 with P < 0.05).

j:spearman negative correlation (> 0.7 with P < 0.05).

k:the reciprocal of the sum of the distances from a node to all other nodes.

l:Refers to the ratio of the shortest path passing through a point and connecting the two points to the total number of shortest path lines between the two points in the network.

**Table S3.**Keystone taxa populations of healthy and disease rhizosphere microbial co-occurrence networks.

| **Number of ASV keystone taxa** | **Group** | **Network type** |
| --- | --- | --- |
| 22 | Healthy | fungal-bacterial inter-kingdom network |
| 81 | Disease |  |
| 49 | Healthy | Bacterial intra-kingdom networks |
| 69 | Disease |  |
| 34 | Healthy | Fungal intra-kingdom networks |
| 28 | Disease |  |

**Table.S4** Differential expression analysis (KOs) of the disease and healthy samples at KEGG pathway.

| **Entry** | **Level** | **Category1** | **Category2** | **Category3** | **Pathway** | **Description** |
| --- | --- | --- | --- | --- | --- | --- |
| K00197 | Enriched | 1. Metabolism | 1.2 Energy metabolism | Methane metabolism | ko00680 | cdhE, acsC; acetyl-CoA decarbonylase/synthase, CODH/ACS complex subunit gamma [EC:2.1.1.245] |
| K00202 | Enriched | 1. Metabolism | 1.2 Energy metabolism | Methane metabolism | ko00680 | fwdC, fmdC; formylmethanofuran dehydrogenase subunit C [EC:1.2.7.12] |
| K00201 | Enriched | 1. Metabolism | 1.2 Energy metabolism | Methane metabolism | ko00680 | fwdB, fmdB; formylmethanofuran dehydrogenase subunit B [EC:1.2.7.12] |
| K00584 | Enriched | 1. Metabolism | 1.2 Energy metabolism | Methane metabolism | ko00680 | mtrH; tetrahydromethanopterin S-methyltransferase subunit H [EC:2.1.1.86] |
| K00194 | Enriched | 1. Metabolism | 1.2 Energy metabolism | Methane metabolism | ko00680 | cdhD, acsD; acetyl-CoA decarbonylase/synthase, CODH/ACS complex subunit delta [EC:2.1.1.245] |
| K00198 | Enriched | 1. Metabolism | 1.11 Xenobiotics biodegradation and metabolism | Nitrotoluene degradation | ko00633 | cooS, acsA; anaerobic carbon-monoxide dehydrogenase catalytic subunit [EC:1.2.7.4] |
| K00171 | Enriched | 1. Metabolism | 1.1 Carbohydrate metabolism | Glycolysis / Gluconeogenesis | ko00010 | porD; pyruvate ferredoxin oxidoreductase delta subunit [EC:1.2.7.1] |
| K00200 | Enriched | 1. Metabolism | 1.2 Energy metabolism | Methane metabolism | ko00680 | fwdA, fmdA; formylmethanofuran dehydrogenase subunit A [EC:1.2.7.12] |
| K00878 | Enriched | 1. Metabolism | 1.8 Metabolism of cofactors and vitamins | Thiamine metabolism | ko00730 | thiM; hydroxyethylthiazole kinase [EC:2.7.1.50] |
| K01575 | Enriched | 1. Metabolism | 1.1 Carbohydrate metabolism | Butanoate metabolism | ko00650 | alsD, budA, aldC; acetolactate decarboxylase [EC:4.1.1.5] |
| K00196 | Enriched | 1. Metabolism | 1.11 Xenobiotics biodegradation and metabolism | Nitrotoluene degradation | ko00633 | cooF; anaerobic carbon-monoxide dehydrogenase iron sulfur subunit |
| K02008 | Enriched | 3. Environmental Information Processing | 3.1 Membrane transport | ABC transporters | ko02010 | cbiQ; cobalt/nickel transport system permease protein |
| K00230 | Enriched | 1. Metabolism | 1.8 Metabolism of cofactors and vitamins | Porphyrin and chlorophyll metabolism | ko00860 | hemG; menaquinone-dependent protoporphyrinogen oxidase [EC:1.3.5.3] |
| K01060 | Enriched | 1. Metabolism | 1.10 Biosynthesis of other secondary metabolites | Penicillin and cephalosporin biosynthesis | ko00311 | cah; cephalosporin-C deacetylase [EC:3.1.1.41] |
| K02007 | Enriched | 3. Environmental Information Processing | 3.1 Membrane transport | ABC transporters | ko02010 | cbiM; cobalt/nickel transport system permease protein |
| K00172 | Enriched | 1. Metabolism | 1.1 Carbohydrate metabolism | Glycolysis / Gluconeogenesis | ko00010 | porG; pyruvate ferredoxin oxidoreductase gamma subunit [EC:1.2.7.1] |
| K01058 | Enriched | 1. Metabolism | 1.3 Lipid metabolism | Ether lipid metabolism | ko00565 | pldA; phospholipase A1/A2 [EC:3.1.1.32 3.1.1.4] |
| K01734 | Enriched | 1. Metabolism | 1.1 Carbohydrate metabolism | Propanoate metabolism | ko00640 | mgsA; methylglyoxal synthase [EC:4.2.3.3] |
| K01666 | Enriched | 1. Metabolism | 1.5 Amino acid metabolism | Phenylalanine metabolism | ko00360 | mhpE; 4-hydroxy 2-oxovalerate aldolase [EC:4.1.3.39] |
| K00768 | Enriched | 1. Metabolism | 1.8 Metabolism of cofactors and vitamins | Porphyrin and chlorophyll metabolism | ko00860 | E2.4.2.21, cobU, cobT; nicotinate-nucleotide--dimethylbenzimidazole phosphoribosyltransferase [EC:2.4.2.21] |
| K01442 | Enriched | 1. Metabolism | 1.3 Lipid metabolism | Primary bile acid biosynthesis | ko00120 | cbh; choloylglycine hydrolase [EC:3.5.1.24] |
| K00170 | Enriched | 1. Metabolism | 1.1 Carbohydrate metabolism | Glycolysis / Gluconeogenesis | ko00010 | porB; pyruvate ferredoxin oxidoreductase beta subunit [EC:1.2.7.1] |
| K00177 | Enriched | 1. Metabolism | 1.1 Carbohydrate metabolism | Citrate cycle (TCA cycle) | ko00020 | korC, oorC; 2-oxoglutarate ferredoxin oxidoreductase subunit gamma [EC:1.2.7.3] |
| K02793 | Enriched | 1. Metabolism | 1.1 Carbohydrate metabolism | Fructose and mannose metabolism | ko00051 | manXa; mannose PTS system EIIA component [EC:2.7.1.191] |
| K00169 | Enriched | 1. Metabolism | 1.1 Carbohydrate metabolism | Glycolysis / Gluconeogenesis | ko00010 | porA; pyruvate ferredoxin oxidoreductase alpha subunit [EC:1.2.7.1] |
| K00176 | Enriched | 1. Metabolism | 1.1 Carbohydrate metabolism | Citrate cycle (TCA cycle) | ko00020 | korD, oorD; 2-oxoglutarate ferredoxin oxidoreductase subunit delta [EC:1.2.7.3] |
| K01077 | Enriched | 1. Metabolism | 1.8 Metabolism of cofactors and vitamins | Thiamine metabolism | ko00730 | E3.1.3.1, phoA, phoB; alkaline phosphatase [EC:3.1.3.1] |
| K01624 | Enriched | 1. Metabolism | 1.1 Carbohydrate metabolism | Glycolysis / Gluconeogenesis | ko00010 | FBA, fbaA; fructose-bisphosphate aldolase, class II [EC:4.1.2.13] |
| K02044 | Enriched | 3. Environmental Information Processing | 3.1 Membrane transport | ABC transporters | ko02010 | phnD; phosphonate transport system substrate-binding protein |
| K01579 | Enriched | 1. Metabolism | 1.6 Metabolism of other amino acids | beta-Alanine metabolism | ko00410 | panD; aspartate 1-decarboxylase [EC:4.1.1.11] |
| K01486 | Enriched | 1. Metabolism | 1.4 Nucleotide metabolism | Purine metabolism | ko00230 | ade; adenine deaminase [EC:3.5.4.2] |
| K02224 | Enriched | 1. Metabolism | 1.8 Metabolism of cofactors and vitamins | Porphyrin and chlorophyll metabolism | ko00860 | cobB-cbiA; cobyrinic acid a,c-diamide synthase [EC:6.3.5.9 6.3.5.11] |
| K01843 | Enriched | 1. Metabolism | 1.5 Amino acid metabolism | Lysine degradation | ko00310 | kamA; lysine 2,3-aminomutase [EC:5.4.3.2] |
| K02227 | Enriched | 1. Metabolism | 1.8 Metabolism of cofactors and vitamins | Porphyrin and chlorophyll metabolism | ko00860 | cbiB, cobD; adenosylcobinamide-phosphate synthase [EC:6.3.1.10] |
| K01804 | Enriched | 1. Metabolism | 1.1 Carbohydrate metabolism | Pentose and glucuronate interconversions | ko00040 | araA; L-arabinose isomerase [EC:5.3.1.4] |
| K02232 | Enriched | 1. Metabolism | 1.8 Metabolism of cofactors and vitamins | Porphyrin and chlorophyll metabolism | ko00860 | cobQ, cbiP; adenosylcobyric acid synthase [EC:6.3.5.10] |
| K02110 | Enriched | 1. Metabolism | 1.2 Energy metabolism | Oxidative phosphorylation | ko00190 | ATPF0C, atpE; F-type H+-transporting ATPase subunit c |
| K01039 | Enriched | 1. Metabolism | 1.11 Xenobiotics biodegradation and metabolism | Styrene degradation | ko00643 | gctA; glutaconate CoA-transferase, subunit A [EC:2.8.3.12] |
| K01834 | Enriched | 1. Metabolism | 1.1 Carbohydrate metabolism | Glycolysis / Gluconeogenesis | ko00010 | PGAM, gpmA; 2,3-bisphosphoglycerate-dependent phosphoglycerate mutase [EC:5.4.2.11] |
| K01912 | Enriched | 1. Metabolism | 1.5 Amino acid metabolism | Phenylalanine metabolism | ko00360 | paaK; phenylacetate-CoA ligase [EC:6.2.1.30] |
| K01040 | Enriched | 1. Metabolism | 1.11 Xenobiotics biodegradation and metabolism | Styrene degradation | ko00643 | gctB; glutaconate CoA-transferase, subunit B [EC:2.8.3.12] |
| K00297 | Enriched | 1. Metabolism | 1.8 Metabolism of cofactors and vitamins | One carbon pool by folate | ko00670 | metF, MTHFR; methylenetetrahydrofolate reductase (NADPH) [EC:1.5.1.20] |
| K01662 | Enriched | 1. Metabolism | 1.8 Metabolism of cofactors and vitamins | Thiamine metabolism | ko00730 | dxs; 1-deoxy-D-xylulose-5-phosphate synthase [EC:2.2.1.7] |
| K00688 | Enriched | 5. Organismal Systems | 5.2 Endocrine system | Glucagon signaling pathway | ko04922 | PYG, glgP; glycogen phosphorylase [EC:2.4.1.1] |
| K01996 | Enriched | 3. Environmental Information Processing | 3.1 Membrane transport | ABC transporters | ko02010 | livF; branched-chain amino acid transport system ATP-binding protein |
| K02946 | Enriched | 2. Genetic Information Processing | 2.2 Translation | Ribosome | ko03010 | RP-S10, MRPS10, rpsJ; small subunit ribosomal protein S10 |
| K00602 | Enriched | 1. Metabolism | 1.4 Nucleotide metabolism | Purine metabolism | ko00230 | purH; phosphoribosylaminoimidazolecarboxamide formyltransferase / IMP cyclohydrolase [EC:2.1.2.3 3.5.4.10] |
| K01997 | Enriched | 3. Environmental Information Processing | 3.1 Membrane transport | ABC transporters | ko02010 | livH; branched-chain amino acid transport system permease protein |
| K01995 | Enriched | 3. Environmental Information Processing | 3.1 Membrane transport | ABC transporters | ko02010 | livG; branched-chain amino acid transport system ATP-binding protein |
| K01998 | Enriched | 3. Environmental Information Processing | 3.1 Membrane transport | ABC transporters | ko02010 | livM; branched-chain amino acid transport system permease protein |
| K02040 | Enriched | 3. Environmental Information Processing | 3.1 Membrane transport | ABC transporters | ko02010 | pstS; phosphate transport system substrate-binding protein |
| K01921 | Enriched | 1. Metabolism | 1.6 Metabolism of other amino acids | D-Alanine metabolism | ko00473 | ddl; D-alanine-D-alanine ligase [EC:6.3.2.4] |
| K00134 | Enriched | 1. Metabolism | 1.1 Carbohydrate metabolism | Glycolysis / Gluconeogenesis | ko00010 | GAPDH, gapA; glyceraldehyde 3-phosphate dehydrogenase [EC:1.2.1.12] |
| K00334 | Enriched | 1. Metabolism | 1.2 Energy metabolism | Oxidative phosphorylation | ko00190 | nuoE; NADH-quinone oxidoreductase subunit E [EC:7.1.1.2] |
| K02358 | Enriched | 5. Organismal Systems | 5.10 Environmental adaptation | Plant-pathogen interaction | ko04626 | tuf, TUFM; elongation factor Tu |
| K00873 | Enriched | 1. Metabolism | 1.1 Carbohydrate metabolism | Glycolysis / Gluconeogenesis | ko00010 | PK, pyk; pyruvate kinase [EC:2.7.1.40] |
| K00927 | Enriched | 1. Metabolism | 1.1 Carbohydrate metabolism | Glycolysis / Gluconeogenesis | ko00010 | PGK, pgk; phosphoglycerate kinase [EC:2.7.2.3] |
| K00606 | Enriched | 1. Metabolism | 1.8 Metabolism of cofactors and vitamins | Pantothenate and CoA biosynthesis | ko00770 | panB; 3-methyl-2-oxobutanoate hydroxymethyltransferase [EC:2.1.2.11] |
| K00790 | Enriched | 1. Metabolism | 1.1 Carbohydrate metabolism | Amino sugar and nucleotide sugar metabolism | ko00520 | murA; UDP-N-acetylglucosamine 1-carboxyvinyltransferase [EC:2.5.1.7] |
| K01951 | Enriched | 1. Metabolism | 1.4 Nucleotide metabolism | Purine metabolism | ko00230 | guaA, GMPS; GMP synthase (glutamine-hydrolysing) [EC:6.3.5.2] |
| K01006 | Enriched | 1. Metabolism | 1.1 Carbohydrate metabolism | Pyruvate metabolism | ko00620 | ppdK; pyruvate, orthophosphate dikinase [EC:2.7.9.1] |
| K00600 | Enriched | 1. Metabolism | 1.5 Amino acid metabolism | Glycine, serine and threonine metabolism | ko00260 | glyA, SHMT; glycine hydroxymethyltransferase [EC:2.1.2.1] |
| K01940 | Enriched | 1. Metabolism | 1.5 Amino acid metabolism | Arginine biosynthesis | ko00220 | argG, ASS1; argininosuccinate synthase [EC:6.3.4.5] |
| K00789 | Enriched | 1. Metabolism | 1.5 Amino acid metabolism | Cysteine and methionine metabolism | ko00270 | metK; S-adenosylmethionine synthetase [EC:2.5.1.6] |
| K00764 | Enriched | 1. Metabolism | 1.4 Nucleotide metabolism | Purine metabolism | ko00230 | purF, PPAT; amidophosphoribosyltransferase [EC:2.4.2.14] |
| K01887 | Enriched | 2. Genetic Information Processing | 2.2 Translation | Aminoacyl-tRNA biosynthesis | ko00970 | RARS, argS; arginyl-tRNA synthetase [EC:6.1.1.19] |
| K01868 | Enriched | 2. Genetic Information Processing | 2.2 Translation | Aminoacyl-tRNA biosynthesis | ko00970 | TARS, thrS; threonyl-tRNA synthetase [EC:6.1.1.3] |
| K01689 | Enriched | 1. Metabolism | 1.1 Carbohydrate metabolism | Glycolysis / Gluconeogenesis | ko00010 | ENO, eno; enolase [EC:4.2.1.11] |
| K02171 | Depleted | 6. Human Diseases | 6.11 Drug resistance: antimicrobial | beta-Lactam resistance | ko01501 | blaI; BlaI family transcriptional regulator, penicillinase repressor |
| K01322 | Depleted | 5. Organismal Systems | 5.2 Endocrine system | Renin-angiotensin system | ko04614 | PREP; prolyl oligopeptidase [EC:3.4.21.26] |
| K01283 | Depleted | 5. Organismal Systems | 5.2 Endocrine system | Renin-angiotensin system | ko04614 | ACE, CD143; peptidyl-dipeptidase A [EC:3.4.15.1] |
| K02054 | Depleted | 4. Cellular Processes | 4.4 Cellular community - prokaryotes | Quorum sensing | ko02024 | ABC.SP.P1; putative spermidine/putrescine transport system permease protein |
| K02651 | Depleted | 4. Cellular Processes | 4.2 Cell growth and death | Cell cycle - Caulobacter | ko04112 | flp, pilA; pilus assembly protein Flp/PilA |
| K01247 | Depleted | 2. Genetic Information Processing | 2.4 Replication and repair | Base excision repair | ko03410 | alkA; DNA-3-methyladenine glycosylase II [EC:3.2.2.21] |
| K00974 | Depleted | 2. Genetic Information Processing | 2.2 Translation | RNA transport | ko03013 | cca; tRNA nucleotidyltransferase (CCA-adding enzyme) [EC:2.7.7.72 3.1.3.- 3.1.4.-] |
| K02549 | Depleted | 1. Metabolism | 1.8 Metabolism of cofactors and vitamins | Ubiquinone and other terpenoid-quinone biosynthesis | ko00130 | menC; O-succinylbenzoate synthase [EC:4.2.1.113] |
| K01113 | Depleted | 1. Metabolism | 1.8 Metabolism of cofactors and vitamins | Folate biosynthesis | ko00790 | phoD; alkaline phosphatase D [EC:3.1.3.1] |
| K00799 | Depleted | 1. Metabolism | 1.6 Metabolism of other amino acids | Glutathione metabolism | ko00480 | GST, gst; glutathione S-transferase [EC:2.5.1.18] |
| K01476 | Depleted | 1. Metabolism | 1.5 Amino acid metabolism | Arginine biosynthesis | ko00220 | E3.5.3.1, rocF, arg; arginase [EC:3.5.3.1] |
| K00316 | Depleted | 1. Metabolism | 1.5 Amino acid metabolism | Arginine and proline metabolism | ko00330 | spdH; spermidine dehydrogenase [EC:1.5.99.6] |
| K01426 | Depleted | 1. Metabolism | 1.5 Amino acid metabolism | Arginine and proline metabolism | ko00330 | E3.5.1.4, amiE; amidase [EC:3.5.1.4] |
| K01556 | Depleted | 1. Metabolism | 1.5 Amino acid metabolism | Tryptophan metabolism | ko00380 | KYNU, kynU; kynureninase [EC:3.7.1.3] |
| K00456 | Depleted | 1. Metabolism | 1.5 Amino acid metabolism | Cysteine and methionine metabolism | ko00270 | CDO1; cysteine dioxygenase [EC:1.13.11.20] |
| K01474 | Depleted | 1. Metabolism | 1.5 Amino acid metabolism | Arginine and proline metabolism | ko00330 | hyuB; N-methylhydantoinase B [EC:3.5.2.14] |
| K00130 | Depleted | 1. Metabolism | 1.5 Amino acid metabolism | Glycine, serine and threonine metabolism | ko00260 | betB, gbsA; betaine-aldehyde dehydrogenase [EC:1.2.1.8] |
| K00657 | Depleted | 1. Metabolism | 1.5 Amino acid metabolism | Arginine and proline metabolism | ko00330 | speG, SAT; diamine N-acetyltransferase [EC:2.3.1.57] |
| K01953 | Depleted | 1. Metabolism | 1.5 Amino acid metabolism | Alanine, aspartate and glutamate metabolism | ko00250 | asnB, ASNS; asparagine synthase (glutamine-hydrolysing) [EC:6.3.5.4] |
| K00499 | Depleted | 1. Metabolism | 1.5 Amino acid metabolism | Glycine, serine and threonine metabolism | ko00260 | CMO; choline monooxygenase [EC:1.14.15.7] |
| K00549 | Depleted | 1. Metabolism | 1.5 Amino acid metabolism | Cysteine and methionine metabolism | ko00270 | metE; 5-methyltetrahydropteroyltriglutamate--homocysteine methyltransferase [EC:2.1.1.14] |
| K00452 | Depleted | 1. Metabolism | 1.5 Amino acid metabolism | Tryptophan metabolism | ko00380 | HAAO; 3-hydroxyanthranilate 3,4-dioxygenase [EC:1.13.11.6] |
| K00453 | Depleted | 1. Metabolism | 1.5 Amino acid metabolism | Tryptophan metabolism | ko00380 | TDO2, kynA; tryptophan 2,3-dioxygenase [EC:1.13.11.11] |
| K01431 | Depleted | 1. Metabolism | 1.4 Nucleotide metabolism | Pyrimidine metabolism | ko00240 | UPB1, pydC; beta-ureidopropionase [EC:3.5.1.6] |
| K01239 | Depleted | 1. Metabolism | 1.4 Nucleotide metabolism | Purine metabolism | ko00230 | iunH; purine nucleosidase [EC:3.2.2.1] |
| K00632 | Depleted | 1. Metabolism | 1.3 Lipid metabolism | Fatty acid degradation | ko00071 | fadA, fadI; acetyl-CoA acyltransferase [EC:2.3.1.16] |
| K00507 | Depleted | 1. Metabolism | 1.3 Lipid metabolism | Biosynthesis of unsaturated fatty acids | ko01040 | SCD, desC; stearoyl-CoA desaturase (Delta-9 desaturase) [EC:1.14.19.1] |
| K00720 | Depleted | 1. Metabolism | 1.3 Lipid metabolism | Sphingolipid metabolism | ko00600 | UGCG; ceramide glucosyltransferase [EC:2.4.1.80] |
| K01028 | Depleted | 1. Metabolism | 1.3 Lipid metabolism | Synthesis and degradation of ketone bodies | ko00072 | E2.8.3.5A, scoA; 3-oxoacid CoA-transferase subunit A [EC:2.8.3.5] |
| K00390 | Depleted | 1. Metabolism | 1.2 Energy metabolism | Sulfur metabolism | ko00920 | cysH; phosphoadenosine phosphosulfate reductase [EC:1.8.4.8 1.8.4.10] |
| K01560 | Depleted | 1. Metabolism | 1.11 Xenobiotics biodegradation and metabolism | Chlorocyclohexane and chlorobenzene degradation | ko00361 | E3.8.1.2; 2-haloacid dehalogenase [EC:3.8.1.2] |
| K01434 | Depleted | 1. Metabolism | 1.10 Biosynthesis of other secondary metabolites | Penicillin and cephalosporin biosynthesis | ko00311 | pac; penicillin G amidase [EC:3.5.1.11] |
| K01637 | Depleted | 1. Metabolism | 1.1 Carbohydrate metabolism | Glyoxylate and dicarboxylate metabolism | ko00630 | E4.1.3.1, aceA; isocitrate lyase [EC:4.1.3.1] |
| K01053 | Depleted | 1. Metabolism | 1.1 Carbohydrate metabolism | Pentose phosphate pathway | ko00030 | E3.1.1.17, gnl, RGN; gluconolactonase [EC:3.1.1.17] |
| K01114 | Depleted | 1. Metabolism | 1.1 Carbohydrate metabolism | Inositol phosphate metabolism | ko00562 | plc; phospholipase C [EC:3.1.4.3] |
| K01647 | Depleted | 1. Metabolism | 1.1 Carbohydrate metabolism | Citrate cycle (TCA cycle) | ko00020 | CS, gltA; citrate synthase [EC:2.3.3.1] |
| K01194 | Depleted | 1. Metabolism | 1.1 Carbohydrate metabolism | Starch and sucrose metabolism | ko00500 | TREH, treA, treF; alpha,alpha-trehalase [EC:3.2.1.28] |
| K01635 | Depleted | 1. Metabolism | 1.1 Carbohydrate metabolism | Galactose metabolism | ko00052 | lacD; tagatose 1,6-diphosphate aldolase [EC:4.1.2.40] |
| K01638 | Depleted | 1. Metabolism | 1.1 Carbohydrate metabolism | Pyruvate metabolism | ko00620 | aceB, glcB; malate synthase [EC:2.3.3.9] |
| K00197 | Enriched | 1. Metabolism | 1.2 Energy metabolism | Methane metabolism | ko00680 | cdhE, acsC; acetyl-CoA decarbonylase/synthase, CODH/ACS complex subunit gamma [EC:2.1.1.245] |
| K00202 | Enriched | 1. Metabolism | 1.2 Energy metabolism | Methane metabolism | ko00680 | fwdC, fmdC; formylmethanofuran dehydrogenase subunit C [EC:1.2.7.12] |
| K00201 | Enriched | 1. Metabolism | 1.2 Energy metabolism | Methane metabolism | ko00680 | fwdB, fmdB; formylmethanofuran dehydrogenase subunit B [EC:1.2.7.12] |
| K00584 | Enriched | 1. Metabolism | 1.2 Energy metabolism | Methane metabolism | ko00680 | mtrH; tetrahydromethanopterin S-methyltransferase subunit H [EC:2.1.1.86] |
| K00194 | Enriched | 1. Metabolism | 1.2 Energy metabolism | Methane metabolism | ko00680 | cdhD, acsD; acetyl-CoA decarbonylase/synthase, CODH/ACS complex subunit delta [EC:2.1.1.245] |
| K00198 | Enriched | 1. Metabolism | 1.11 Xenobiotics biodegradation and metabolism | Nitrotoluene degradation | ko00633 | cooS, acsA; anaerobic carbon-monoxide dehydrogenase catalytic subunit [EC:1.2.7.4] |
| K00171 | Enriched | 1. Metabolism | 1.1 Carbohydrate metabolism | Glycolysis / Gluconeogenesis | ko00010 | porD; pyruvate ferredoxin oxidoreductase delta subunit [EC:1.2.7.1] |
| K00200 | Enriched | 1. Metabolism | 1.2 Energy metabolism | Methane metabolism | ko00680 | fwdA, fmdA; formylmethanofuran dehydrogenase subunit A [EC:1.2.7.12] |
| K00878 | Enriched | 1. Metabolism | 1.8 Metabolism of cofactors and vitamins | Thiamine metabolism | ko00730 | thiM; hydroxyethylthiazole kinase [EC:2.7.1.50] |
| K01575 | Enriched | 1. Metabolism | 1.1 Carbohydrate metabolism | Butanoate metabolism | ko00650 | alsD, budA, aldC; acetolactate decarboxylase [EC:4.1.1.5] |
| K00196 | Enriched | 1. Metabolism | 1.11 Xenobiotics biodegradation and metabolism | Nitrotoluene degradation | ko00633 | cooF; anaerobic carbon-monoxide dehydrogenase iron sulfur subunit |
| K02008 | Enriched | 3. Environmental Information Processing | 3.1 Membrane transport | ABC transporters | ko02010 | cbiQ; cobalt/nickel transport system permease protein |
| K00230 | Enriched | 1. Metabolism | 1.8 Metabolism of cofactors and vitamins | Porphyrin and chlorophyll metabolism | ko00860 | hemG; menaquinone-dependent protoporphyrinogen oxidase [EC:1.3.5.3] |

**Table S5.** The physicochemical properties of the five sites sampled in this study

| **Site** | **pH** | **TN** | **AN** | **AP** | **AK** | **TC** | **OM** | **NH_4_^+^-N** | **NO_3_^-^-N** |
| --- | --- | --- | --- | --- | --- | --- | --- | --- | --- |
| YJ | 4.891±0.12b | 0.246±0.02b | 119.358±1.36b | 8.55±1.84c | 38.605±2.34b | 2.532±0.07b | 4.641±0.12b | 59.197±4.04b | 28.654±2.25b |
| JH | 4.979±0.24b | 0.291±0.08b | 117.11±15.36b | 16.023±2.06b | 43.003±16.03b | 2.926±0.49a | 5.045±0.84b | 77.03±5.47a | 29.697±3.96b |
| NB | 4.997±0.19b | 0.32±0.07a | 122.30±11.50a | 15.044±5.14b | 43.993±15.28a | 2.77±0.39b | 5.465±0.67a | 63.416±8.10b | 32.432±3.45a |
| RA | 5.251±0.19a | 0.239±0.04b | 95.648±7.42c | 6.538±2.34d | 39.94±6.97b | 2.499±0.34b | 5.309±0.58a | 58.998±7.05b | 29.188±2.75b |
| TZ | 5.018±0.26b | 0.292±0.06a | 115.94±7.29b | 28.146±4.02a | 41.89±3.29a | 2.423±0.36b | 5.177±1.22a | 81.731±4.66a | 32.358±4.83a |

Means followed by different letters within columns are significantly different. Data are means ± standard error (SEs) (Duncan test, p < 0.05).YJ, Yongjia City (120°44'14"E, 28°9'38"N), JH, Jinhua City (119°25'10"E, 21°0'51"N), NB, Ningbo City (121°32'54"E, 29°57'50"N), RA, Ruian City (120°44'1"E, 27°50'22"N), TZ, Taizhou City (121°17'19"E, 28°34'6"N). TN, total nitrogen, AN, available nitrogen, AP, available phosphorus, AK, available potassium, TC, total carbon, OM, organic matter, NH_4_^+^-N, ammonium, NO_3_^-^-N, nitrate.
